# Modeling maternal immune activation in 3D ex vivo human fetal brain cerebroids reveals IL-17A-driven disruption of cortical development

**DOI:** 10.1038/s41593-026-02400-2

**Published:** 2026-08-11

**Authors:** Muhammad Z. K. Assir, Mario Yanakiev, Do Hyeon Gim, Sara S. M. Valkila, Paola Muscolino, Peng Liu, Laetitia L. Lecante, Paul A. Fowler, Daniel A. Berg, Eunchai Kang

**Affiliations:** Institute of Medical Sciences, University of Aberdeen, Aberdeen, United Kingdom

**Keywords:** Cellular neuroscience, Cell fate and cell lineage, Developmental neurogenesis, Neuroimmunology, Disease model

## Abstract

Maternal immune activation (MIA) disrupts brain development and increases the risk of neurodevelopmental disorders, yet the mechanisms by which MIA impacts human cortical development remain poorly understood. Here we introduce a three-dimensional ex vivo culture system, termed ‘cerebroids,’ derived from the dorsolateral prefrontal cortex of human fetal brain tissue, which preserves the key developmental processes, cellular diversity and structural integrity of the developing human cortex. Using this model, we show that IL-17A, a cytokine implicated in MIA and neurodevelopmental disorders, induces premature cortical folding, increases cortical thickness and accelerates neurogenesis and neuronal maturation. We reveal that IL-17A substantially dysregulates extracellular-matrix-related pathways, including upregulation of proteoglycans. In neural stem cells, IL-17A directly activates NF-κB signaling, leading to sustained inflammatory responses that contribute to these developmental abnormalities, which are reversed by treatment with the NF-κB pathway inhibitor parthenolide. These findings delineate how IL-17A perturbs human corticogenesis while elucidating the mechanisms underlying brain disruption during MIA.

## Abstract (web summary)

Using cerebroids, a 3D ex vivo model of the human fetal brain, the authors show that IL-17A, a mediator of maternal immune activation linked to neurodevelopmental disorders, disrupts early human cortical development through NF-κB signaling.

## Main

A growing body of evidence from epidemiological studies has demonstrated that maternal immune activation (MIA) significantly increases the risk of neurodevelopmental disorders (NDDs), including autism spectrum disorder (ASD) and schizophrenia, in offspring^1–5^. Animal models of MIA have provided insights into its role in the pathogenesis of NDDs, revealing ASD-related behavioral deficits and structural brain abnormalities^6,7^, such as increased cortical thickness, elevated neuronal numbers^8^ and perturbed cortical layer organization^7^. Despite these findings, the precise cellular and molecular mechanisms by which the inflammatory environment in utero alters the developmental trajectory of the human cortex, leading to such structural and functional abnormalities, remain poorly understood.

Emerging evidence implicates IL-17A as a key mediator of MIA-associated neurodevelopmental risk. Elevated serum IL-17A levels have been shown to correlate with the severity of ASD in humans^9–11^. Genetic studies have also identified amplified copy number variations in the *IL17A* gene among ASD cohorts^12^. Animal studies indicate that IL-17A is a key mediator of MIA, as elevated maternal IL-17A reaches the fetal brain and is sufficient to induce abnormal cortical development, microglial activation and autism-like behaviors in offspring, while its inhibition prevents these MIA-associated abnormalities^1,7,13–16^.

Human pluripotent stem cell (PSC)-derived brain organoids offer a valuable platform for investigating human-specific aspects of developmental disturbances caused by genetic and environmental insults^17^. However, the absence of immune and vascular components in these organoid models restricts their use, particularly in modeling MIA^18^. Ex vivo models using primary human tissue provide a more accurate representation of brain development, but conventional slice cultures are limited by low throughput and scalability issues. To address this critical gap, we have developed a high-throughput three-dimensional (3D) ex vivo culture system, termed ‘cerebroids’, which preserves key developmental processes and structural organization.

Using cerebroids, we examined the impact of IL-17A, a cytokine mediating MIA. IL-17A disrupts corticogenesis by inducing premature folding, increasing cortical thickness and accelerating neuronal differentiation. Multi-omic analyses reveal extracellular matrix (ECM) dysregulation and sustained NF-κB activation in neural stem cells (NSCs), while parthenolide (PTL), an NF-κB inhibitor, rescues these abnormalities, providing mechanistic insight into how MIA perturbs human cortical development.

## Results

### Establishment and validation of a 3D ex vivo culture of human fetal brain

**Fig. 1:**
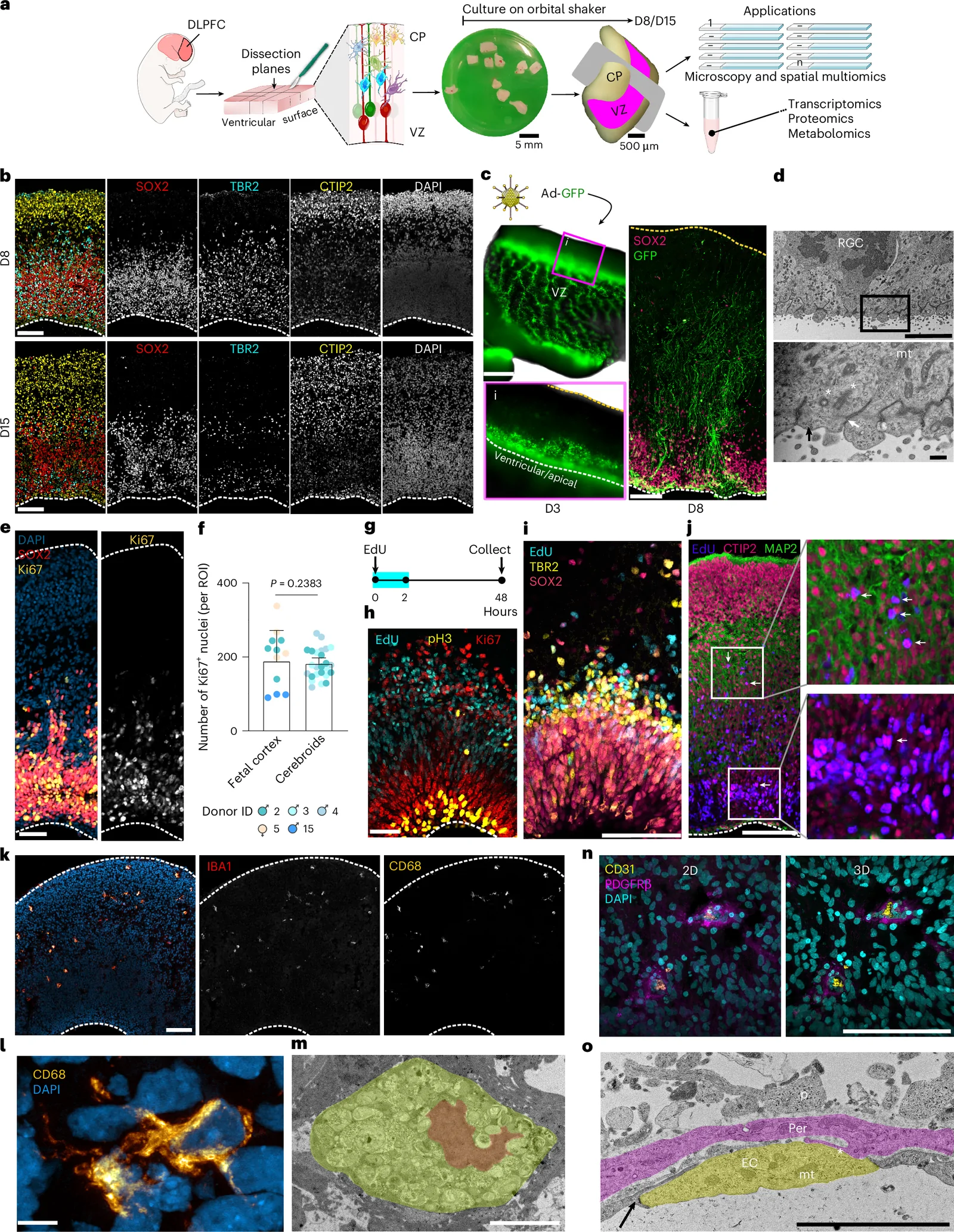
Establishment and validation of a 3D ex vivo culture of human fetal brain. **a**, Schematic illustration of the experimental paradigm. The DLPFC was dissected, cultured and processed for analysis. Illustration was partly generated using Servier Medical Art, provided by Servier, licensed under a Creative Commons license CC BY 4.0. **b**, Representative immunofluorescence images of cerebroids cultured for 8 d and 15 d, showing the layered organization of developing brain tissue. Dotted lines indicate the ventricular surface. **c**, Left: wholemount live image of adenovirus-GFP-infected cerebroids after 3 days in culture. Right: immunofluorescence image of a section from day 8 cerebroids showing GFP^+^ NSCs. **d**, TEM images of day 2 cerebroids illustrating NSCs lining the ventricular surface (top); higher-magnification view of the apical surface (bottom) showing mitochondria (mt), centrioles (*), apical junction (white arrow) and secretory vesicles (black arrow) **e**, Immunofluorescence image of proliferating (Ki67^+^) NSCs in cerebroids cultured for 8 d. **f**, Quantification of the number of Ki67^+^ cells in fetal cortex (*n* = 3 donors) versus day 8 cerebroids (*n* = 9 cerebroids from 3 donors). Data are presented as mean ± s.e.m. Unpaired *t*-test (two-sided). **g**–**j**, EdU pulse–chase experiment on cerebroids. Schematic of the EdU pulse–chase experimental design (**g**), with maximum intensity projection images showing mitotic (pH3^+^), proliferating (Ki67^+^) and EdU^+^ cells (**h**), EdU^+^TBR2^+^ intermediate progenitors (**i**) and EdU^+^CTIP2^+^ and MAP2^+^ neurons (arrows) in cerebroids cultured for 2 d (**j**). **k**, Immunofluorescence images of IBA1^+^ and CD68^+^ microglia in cerebroids cultured for 8 d. **l**, Magnified image of a CD68^+^ microglial cell. **m**, TEM image of a microglial cell, with pseudocoloring of the nucleus (brown) and cell body (yellow) in cerebroids cultured for 8 d. **n**, Immunofluorescence images of PDGFRβ^+^ pericytes and CD31^+^ endothelial cells in cerebroids cultured for 8 d. **o**, TEM image of a blood vessel in day 2 cerebroids, showing endothelial cells (EC), tight junction (arrow), pericytes (Per), pericyte peg-sockets (*), radial glia processes (p) and mitochondria (mt) (also see Extended Data Fig. 2h–j for higher-magnification images of the same). Scale bars: 500 μm (**c** (left)), 100 μm (**b**, **c** (right), **e**, **h**–**k**, **n**), 5 μm (**d** (top), **l**, **m**, **o**) and 500 nm (**d**, bottom). Ad-GFP, adenovirus-GFP; CP, cortical plate; D, day; RGC, radial glial cell; VZ, ventricular zone. Source data

**Extended Data Fig. 1:**
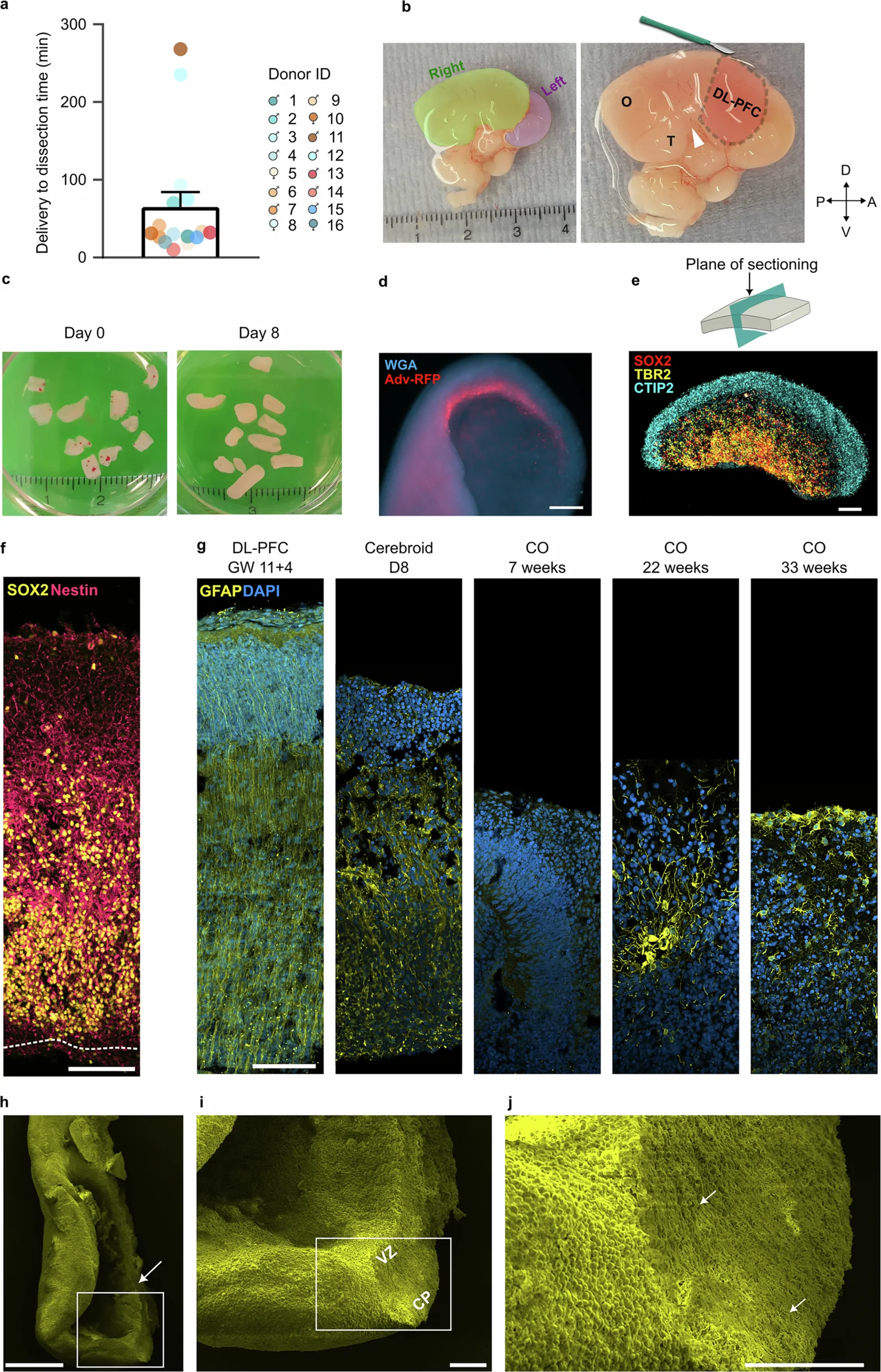
Generation of cerebroids from human dorsolateral prefrontal cortex (DLPFC) tissue and characterization of NSCs in cerebroid cultures. **a**, Time between delivery of the fetus and brain dissection recorded for 14 donors (in minutes). Data is represented as mean ± s.e.m. and each data point represents individual donor (See Supplementary Table 1 for donor details). **b**, Dissected human brain at gestational week (GW) 12. The right hemisphere is highlighted in green and the left hemisphere in purple, with the DLPFC outlined by a dashed line. The occipital (O) and temporal (T) lobes are labelled, and the lateral sulcus is marked by an arrowhead. Brain orientation: posterior (P), anterior (A), dorsal (D), and ventral (V). A ruler (in cm) is shown for scale. For cerebroid culture, DLPFC was identified by the anatomical landmarks shown here and dissected. **c**, Images of cerebroids immediately after dissection (Day 0) and following 8 days in culture (Day 8). Note the increase in the size of the cerebroids after 8 days in culture. **d**, Fluorescence image of Day 8 cerebroid stained with Wheat Germ Agglutinin (WGA), marking glycocalyx and proteoglycans in the ECM, and adenovirus-RFP-labelled neural stem cells (NSCs) lining the ventricular surface. Scale bar, 500μm. **e**, Schematic of the sectioning plane and a fluorescence image of a whole cerebroid section stained for SOX2 (NSCs), TBR2 (IPCs), and CTIP2 (neurons). Scale bars: 100 μm. **f**, Representative fluorescence image of SOX2^+^ and Nestin^+^ NSCs in cerebroid culture. Scale bar, 100 μm. **g**, Fluorescence images showing GFAP-expressing radial glia (RG) in the human DLPFC, day 8 (D8) cerebroids, and cortical organoids (COs) cultured for 7, 22, and 33 weeks. Note the absence of GFAP staining in the NSC layer of 7-week-old COs, while only GFAP^+^ parenchymal astrocytes are observed in 22- and 33-week-old COs. This contrasts with GFAP^+^ processes of NSCs in DL-PFC and D8 cerebroids. Scale bar, 100 μm. **h–j**, Scanning electron microscopy (SEM) images of day 8 cerebroid. (**h**) Low-magnification image showing the cut surface of a cerebroid. (**i**) Higher-magnification image highlighting cytoarchitectural features, including the ventricular zone (VZ) and cortical plate (CP). (**j**) SEM image showing radial glial cell processes (white arrows). Scale bars: 500 μm (**h**), 100 μm (**i**, **j**).

To establish the 3D ex vivo culture system of the human fetal brain, we dissected the dorsolateral prefrontal cortex (DLPFC) from healthy fetal brain tissues at 11–14 gestational weeks, a critical period of active neurogenesis in the developing cortex^19^ and when MIA is most strongly associated with an increased risk of NDDs^3^. The average interval between fetal delivery and brain dissection was 65 min, allowing preservation of tissue integrity (Extended Data Fig. 1a,b; please see Supplementary Table 1 for the donor information). The tissue was cut into approximately 10-mm^2^ pieces, ensuring that each section retained intact architecture from the ventricular surface to the pial surface. These pieces were cultured in a free-floating manner on an orbital shaker (Fig. 1a and Extended Data Fig. 1c,d). This culture method preserves the structural integrity of the tissue and provides versatility for multiple downstream applications, including immunofluorescence staining on cryosections and comprehensive multi-omic analyses (Fig. 1a and Extended Data Fig. 1e).

**Extended Data Fig. 2:**
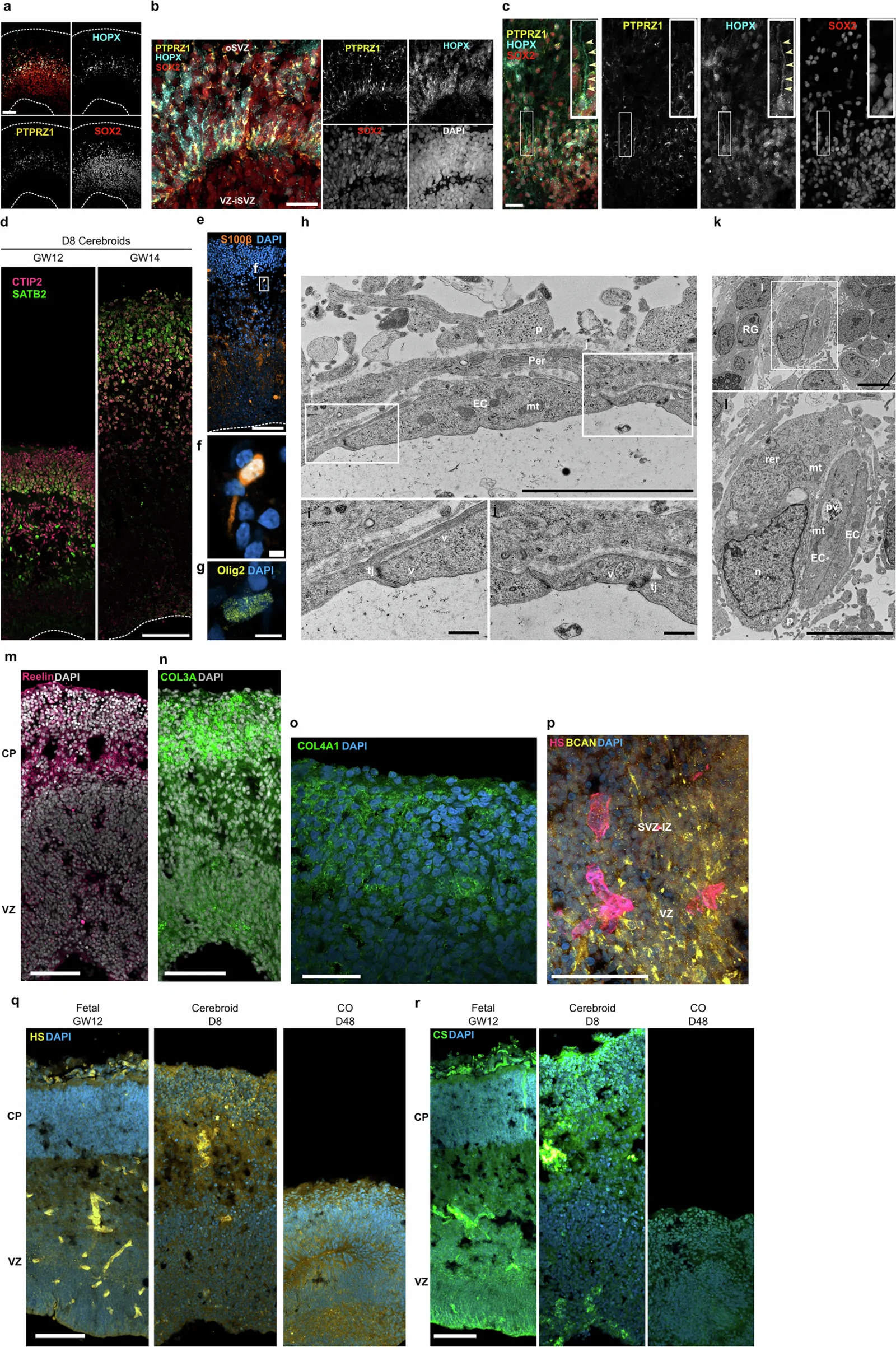
Characterization of oRG, neurons, macroglia progenitors, and vascular structures, and ECM in cerebroid cultures. **a-c**, Representative immunofluorescence images showing outer radial glial cells (oRGs) in the outer subventricular zone (oSVZ) of cerebroids cultured for 8 days. oRGs express the markers PTPRZ1 and HOPX. Yellow arrowheads highlight the HOPX^+^ processes of oRGs. Ventricular zone (VZ) and inner subventricular zone (iSVZ) are indicated. Scale bars: 100 μm (**a**,**b**) and 25 μm (**c**). **d**, Representative immunofluorescence images showing the expression of neuronal markers in Day 8 cerebroids from 12- and 14-week-old fetuses. Neuronal markers include CTIP2 (deep-layer neurons), and SATB2 (late-born upper-layer neurons). Scale bar, 100 μm. **e-g**, Immunofluorescence images of different cell types in cerebroids: S100β^+^ astroglia progenitors (**e**, **f**) and OLIG2^+^ oligodendrocyte precursor cells (OPCs) (**g**). Scale bars: 100 μm (**e**) and 5 μm (**f**, **g**). **h–j** TEM images of vascular structures in Day 2 cerebroids. (**h**) Annotated image from Fig. 1o, showing an endothelial cell (EC) with mitochondria (mt), surrounded by a pericyte (Per) and the end-foot process of a radial glial cell (RGC, p). Zoomed-in views (**i**, **j**) show tight junctions (tj) between endothelial cells, forming part of the blood-brain barrier, and secretory vesicles (v). Scale bar, 5 μm (**h**), and 500 nm (**i**, **j**). **k**, **l**, TEM images of a vascular unit lacking a lumen, showing a radial glial cell (RG), endothelial cell (EC), and pericyte (Per) with nucleus (n). Both endothelial cells and pericytes are rich in mitochondria (mt). The endothelial cell contains a phagocytic vesicle (pv), while the pericyte shows a high content of rough endoplasmic reticulum (rer). A peg-socket junction (*) between the pericyte and endothelial cell is also visible. Scale bar, 5 μm. **m-p**, Fluorescence images show key ECM components in day 8 cerebroids including reelin (**m**), collagen III alpha (**n**), collagen IV alpha 1 (**o**) and brevican (BCAN) (**p**)**. q**, Representative fluorescence images of Heparan Sulfate (HS) staining in a GW12 DLPFC section, an 8-day cultured cerebroid, and a 48-day-old cortical organoid (CO). HS fluorescence intensities are higher and more abundant in DLPFC and cerebroids compared to CO. Abbreviations: VZ, ventricular zone; SVZ, subventricular zone; IZ, intermediate zone. **r**, Representative fluorescence images of Chondroitin Sulfate (CS) staining in a GW12 DLPFC section, an 8-day cultured cerebroid, and a 48-day-old CO. In contrast to CO, cerebroids show greater abundance of CS mimicking expression pattern in DLPFC. Scale bars, 100 μm.

The cytoarchitecture and apical–basal polarity of cells in the developing brain, particularly NSCs, are critical for the proper developmental trajectory of the cortex^19^. Cerebroids cultured for 8 days and 15 days retained the organized layers of SOX2^+^ NSCs, TBR2^+^ intermediate progenitor cells (IPCs) and CTIP2^+^ neurons (Fig. 1b). The proper apical–basal polarity of NSCs and the integrity of their processes were confirmed by adenovirus-mediated GFP labeling of NSCs (Fig. 1c), Nestin and GFAP immunostaining (Extended Data Fig. 1f,g), as well as scanning electron microscopy (SEM) imaging (Extended Data Fig. 1h–j). Notably, unlike cortical organoids derived from human induced PSCs (iPSCs), the expression pattern of GFAP in NSCs within cerebroids was comparable to that of acutely fixed fetal tissue (Extended Data Fig. 1g). The integrity of apical junctions in NSCs was verified by transmission electron microscopy (TEM) (Fig. 1d). Optimal culture conditions that ensure the proper proliferation rate of NSCs are crucial for maintaining the polarity between the ventricular and cortical surfaces^20^. In cerebroids, NSCs exhibited a proliferative capacity comparable to that of acutely fixed fetal cortex, as demonstrated by Ki67 staining (Fig. 1e,f), highlighting that the cerebroid culture media is optimal for maintaining the appropriate proliferation rate of NSCs. Furthermore, the capacity of NSCs to generate IPCs and neurons was confirmed through 5′-ethynyl-2′-deoxyuridine (EdU) pulse–chase experiments (Fig. 1g–j). Mitotic nuclei of NSCs (pH3^+^) were also observed near the ventricular surface, mirroring the behavior of NSCs undergoing interkinetic nuclear migration during cell division in the developing cortex (Fig. 1h). Next, we verified the presence of different cell types in cerebroids including HOPX^+^ or PTPRZ1^+^ outer radial glial cells, CTIP2^+^ or SATB2^+^ neurons, S100β^+^ or Olig2^+^ macroglia precursor cells and IBA1^+^/CD68^+^ microglia (Extended Data Fig. 2a–g and Fig. 1k–m). We also confirmed the presence of vascular structures containing endothelial cells and pericytes (Fig. 1n,o and Extended Data Fig. 2h–l).

The ECM creates a dynamic environment that facilitates interactions among various cell types by modulating the diffusion and distribution of signaling molecules. It is also involved in the regulation of central developmental processes, such as cell proliferation, differentiation and migration^20^. We confirmed that cerebroids express key components of the ECM in the developing cortex, such as heparan sulfate, brevican (BCAN), chondroitin sulfate, collagens and ECM-associated protein Reelin (Extended Data Fig. 2m–r). Importantly, cerebroids, in contrast to iPSC-derived cortical organoids, showed expression patterns of heparan sulfate and chondroitin sulfate that were comparable to acutely fixed fetal tissue (Extended Data Fig. 2q,r).

Together, these findings demonstrate that cerebroids offer a robust platform that preserves key cellular architecture, along with immune, vascular and essential ECM components, enabling the study of both healthy neurodevelopment and disruptions caused by environmental factors.

### Abnormal cortical development mediated by IL-17A

**Fig. 2:**
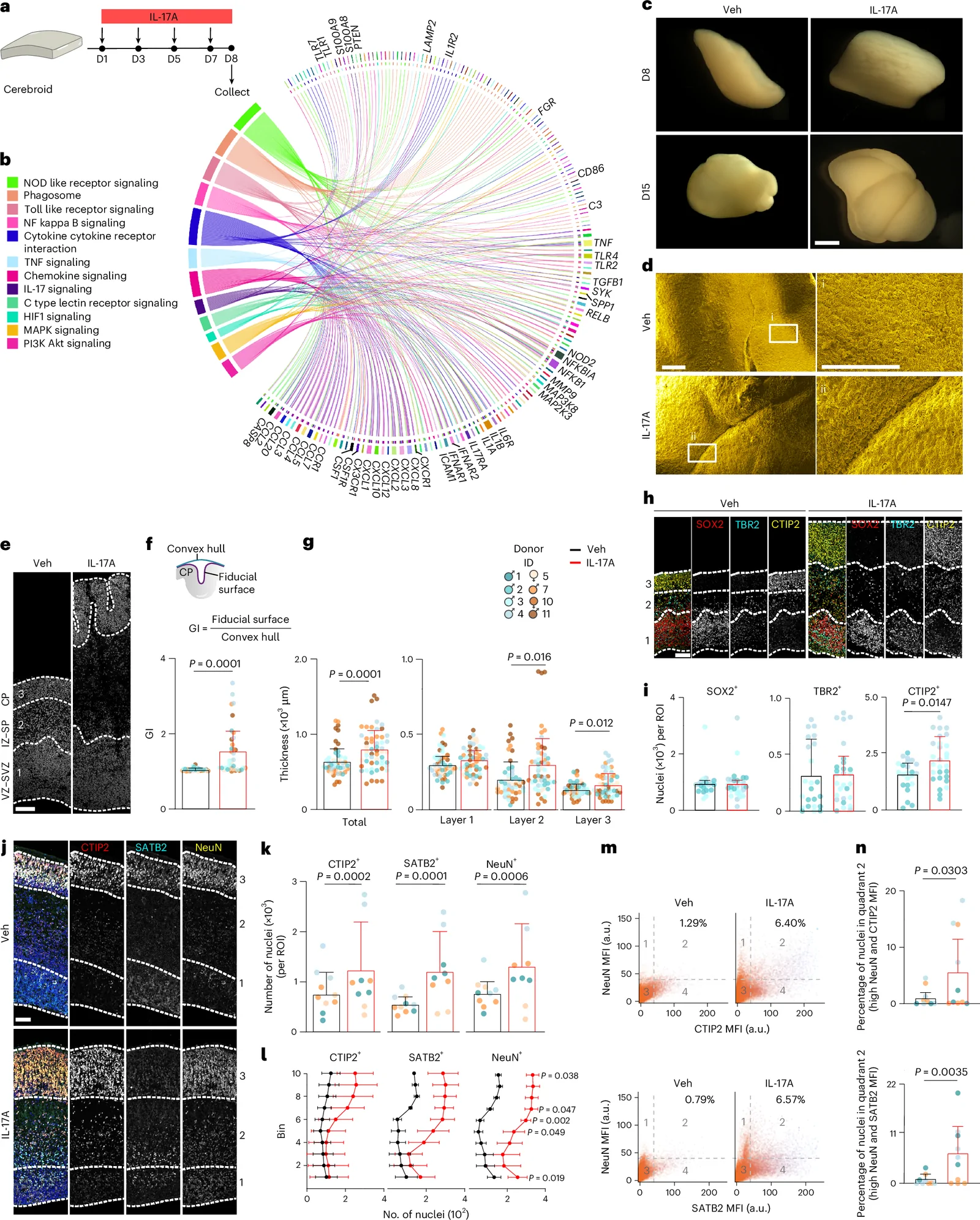
IL-17A treatment promotes cortical folding and enhances neuronal numbers and maturation in cerebroid culture. **a**, Schematic illustration of the experimental paradigm for IL-17A treatment in cerebroid culture. **b**, Chord plot showing upregulated genes (right) and their associated KEGG pathways (left) in microglia following IL-17A treatment. **c**, Representative images of cerebroids cultured for 8 days (D8) and 15 days (D15), demonstrating morphological changes. **d**, SEM images of the cortical surfaces in vehicle- and IL-17A-treated cerebroids, demonstrating structural difference. **e**, DAPI-stained cross-sections of vehicle- and IL-17A-treated cerebroids, showing alterations in cortical architecture. The layers shown are ventricular and subventricular zones (VZ–SVZ, layer 1), intermediate zone and subplate (IZ–SP, layer 2) and cortical plate (CP, layer 3). **f**, Schematic illustration of GI calculation (top) and quantification (bottom), demonstrating increased cortical folding in IL-17A- compared with vehicle-treated control cerebroids. Two-way ANOVA (two-sided). *n* = 18 cerebroids from 7 donors in each group. **g**, Quantification of total and layer-specific cortical thickness (µm) in vehicle- and IL-17A-treated cerebroids. Two-way ANOVA (two-sided). *n* = 18 cerebroids from 7 donors in each group. **h**, Representative immunofluorescence images of NSCs (SOX2), IPCs (TBR2) and neurons (CTIP2) in vehicle- and IL-17A-treated cerebroids, demonstrating increased number of CTIP2^+^ neurons. **i**, Quantification of NSC, IPC and neuronal numbers from **h** in vehicle- and IL-17A-treated cerebroids. Two-way ANOVA (two-sided). *n* = 9 cerebroids from 3 donors in each group. **j**,**k**, Representative immunofluorescence images (**j**) and quantification (**k**) of neurons expressing CTIP2, SATB2 and NeuN in vehicle- and IL-17A-treated cerebroids. Two-way ANOVA (two-sided). *n* = 10 (vehicle) and *n* = 9 (IL-17A) cerebroids from 4 donors. **l**, Quantification of neuronal numbers across cortical layers (bins 1–10, from the ventricular to pial surface), showing spatial distribution in vehicle- and IL-17A-treated cerebroids. Two-way ANOVA. *n* = 3 cerebroids from 3 donors in each group. **m**, Scatter plots displaying MFI (arbitrary units) of NeuN, CTIP2 and SATB2 across nuclei per ROI in vehicle- and IL-17A-treated cerebroids. **n**, Quantification of percentage of CTIP2^+^ and SATB2^+^ neuronal populations with high NeuN expression per ROI, corresponding to **m**. Two-way ANOVA (two-sided). *n* = 9 (vehicle) and *n* = 10 (IL-17A) cerebroids from 3 donors. For **f**, **g**, **i**, **k**, **l** and **n**, data are represented as mean ± s.e.m. Scale bars: 1 mm (**c**) and 100 μm (**d**, **e**, **h**, **j**). Veh, vehicle. Source data

**Extended Data Fig. 3:**
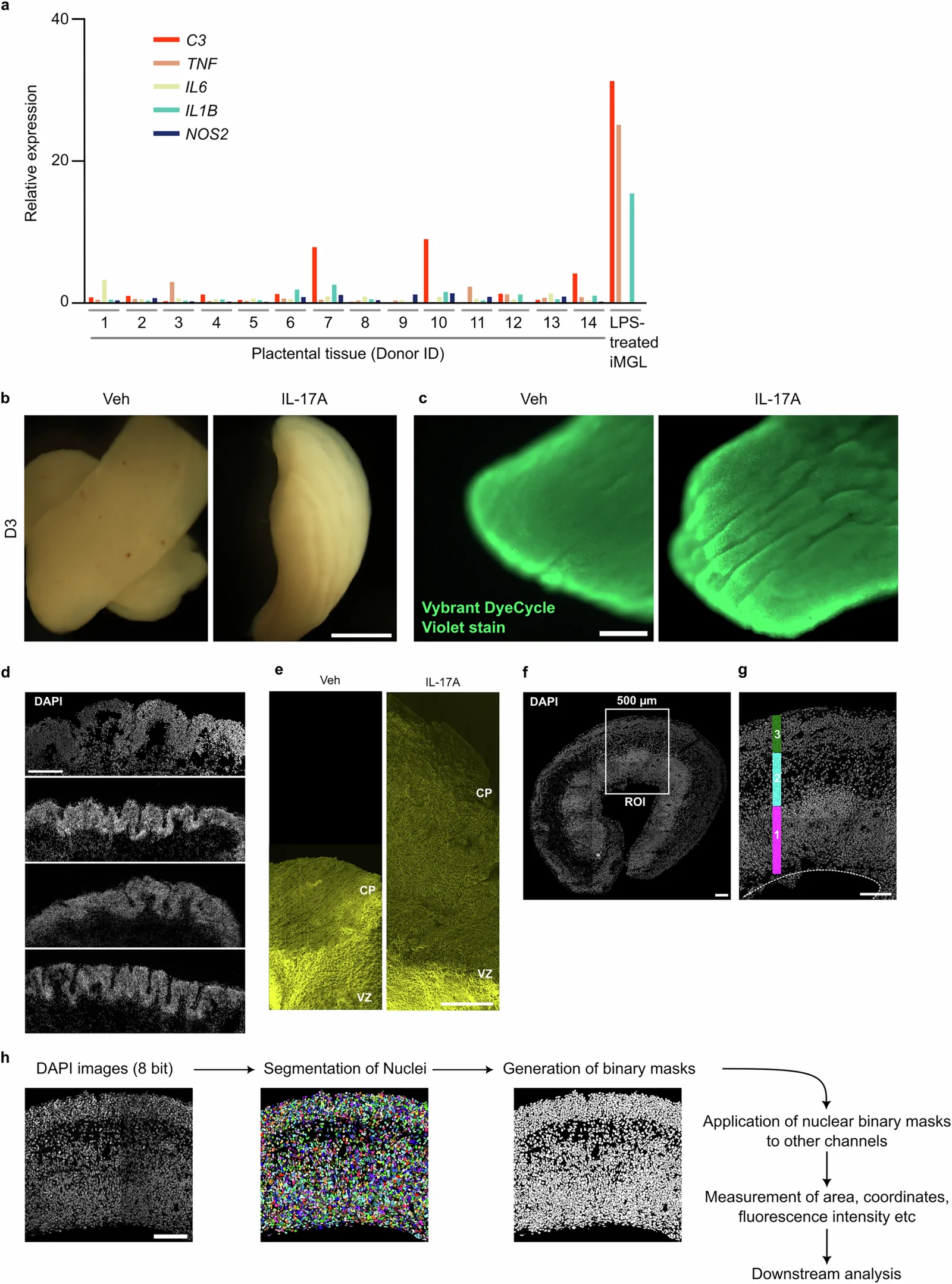
Characterization of IL-17A-induced cellular phenotypes in cerebroids. **a**, qPCR analysis of inflammatory marker expression in placental tissue from respective donors, compared to LPS-treated iPSC-derived microglia like cells (iMGL). **b**, Representative images of vehicle- and IL-17A-treated cerebroids after 3 days of culture. Cortical folding is evident in IL-17A-treated day 3 cerebroid. Scale bar, 1000 μm. **c**, Representative live fluorescence images of vehicle- and IL-17A-treated day 8 cerebroids stained with a nuclear dye (Vybrant DyeCycle Violet). IL-17A-treated cerebroids show abundant and deep folding on the cortical surface in contrast to occasional, shallow folds in vehicle-treated cerebroids. Scale bar, 500 μm. **d**, Examples of various gyrification patterns in Day 8 IL-17A-treated cerebroids, visualized by DAPI staining. Scale bar, 100 μm. **e**, SEM images of cut surfaces of Day 8 vehicle- and IL-17A-treated cerebroids, showing increased thickness in IL-17A-treated cerebroids. Scale bar, 100 μm. **f**, **g**, Image-based quantifications for cell numbers and fluorescence intensities. Representative images of DAPI-stained Day 8 cerebroid sections are shown. (**f**) A fixed-width region of interest (ROI) was drawn through the central part of the cerebroid for image-based quantifications. Scale bar, 100 μm. (**g**) Magnified view of the ROI from **f**. The dotted line represents the ventricular surface. Three layers were identified based on cell densities and immunofluorescence markers: SOX2-rich layer 1 and CTIP2-rich layer 3 (as shown in Fig. 1b). Scale bars, 100 μm. **h**, Schematic of the image analysis pipeline for fluorescence intensity-based analysis (as shown in Fig. 2m-n). 8-bit images of DAPI-stained nuclei were used to segment individual nuclei, followed by the generation of binary masks. These masks were then split to generate ROIs for individual nuclei, and fluorescence intensities were measured across these nuclear ROIs for various channels. Source data

**Extended Data Fig. 4:**
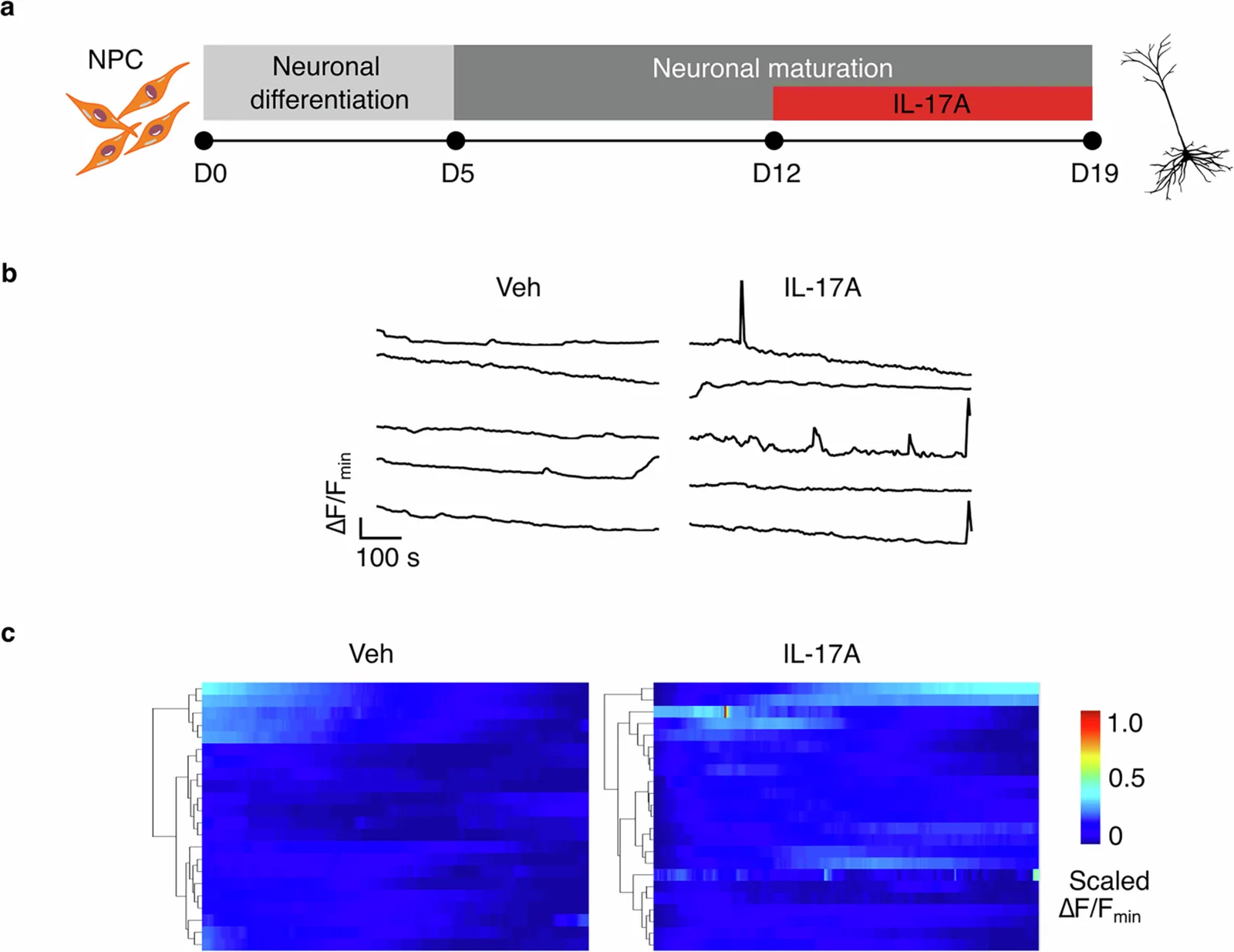
IL-17A-treatment increases calcium transients in 2D cultured iPSC-derived neurons. **a**, Schematic of the experimental paradigm for 2D neuronal culture and IL-17A treatment. iPSC-derived neural progenitor cells (NPCs) were differentiated into forebrain neurons, followed by neuronal maturation. From Day 12 to Day 19, neurons were treated with 10 ng/mL IL-17A every 2 days to assess the effects of IL-17A on neuronal maturity. **b**, Representative calcium transient plots in vehicle- and IL-17A-treated neurons. Each tracing represents the ratio of change in mean fluorescence intensity (MFI) from the minimum MFI (∆F) to the minimum MFI (F_min_) over time. IL-17A-treated neurons exhibit increased amplitude and frequency of spontaneous calcium transient activity compared to vehicle-treated controls. **c**, Heatmaps of calcium transients in vehicle- and IL-17A-treated neurons. Each row represents an individual neuron, while each column represents the time scale. Colours indicate the scaled ∆F/F_min_ values over time, highlighting the heightened calcium activity in IL-17A-treated neurons. Source data

To further explore the impact of IL-17A during MIA in cortical development, we utilized cerebroids from gestational week 11–14 fetal brain tissues. Microglia/brain-associated macrophages (BAMs), as the resident immune cells of the developing cortex, are key responders to inflammatory signals and thus serve as a critical readout of IL-17A activity. To determine whether IL-17A treatment was sufficient to activate neuroinflammatory responses in the cerebroid model, we performed RNA sequencing (RNA-seq) of microglia/BAMs isolated from cerebroids. Transcriptomic analysis revealed that IL-17A treatment in culture activated various inflammation-related pathways, such as TLR, TNF and NF-κB signaling (Fig. 2a,b). All donors used in this study exhibited comparable baseline inflammatory states, as determined by transcriptomic analysis of inflammatory markers in placental tissue (Extended Data Fig. 3a). IL-17A-treated cerebroids showed premature induction of cortical folding at day 8 ex vivo and the folding phenotype became more prominent at day 15 ex vivo (Fig. 2c and Extended Data Fig. 3b,c). SEM imaging further confirmed alterations in the cortical surface structure of IL-17A-treated cerebroids (Fig. 2d). To measure the degree of folding, we calculated the gyrification index (GI), the ratio of the fiducial surface length to the convex hull length, and it was found that IL-17A-treated cerebroids exhibited increased GI (Fig. 2e,f and Extended Data Fig. 3d). Moreover, IL-17A-treated cerebroids exhibited a significant increase in overall thickness, which was observed across cortical layers 2 and 3 (Fig. 2e,g and Extended Data Fig. 3e–g). Particularly, the intermediate zone and subplate (layer 2) showed reduction in cell density, suggesting a potential expansion of ECM in IL-17A-treated cerebroids (Fig. 2e). Next, we investigated which cell types contributed to the increased thickness. The number of CTIP2^+^ deep layer neurons was significantly higher in IL-17A-treated cerebroids, while no significant difference was observed in the number of SOX2^+^ NSCs or TBR2^+^ IPCs (Fig. 2h,i and Extended Data Fig. 3h). Additionally, IL-17A treatment increased the number of SATB2^+^ upper-layer neurons and NeuN^+^ mature neurons (Fig. 2j–l). IL-17A treatment also resulted in an elevated proportion of CTIP2^+^ and SATB2^+^ cells expressing higher levels of NeuN (Fig. 2m,n), suggesting accelerated neuronal maturation in IL-17A-treated cerebroids. The increased number of excitatory neurons induced by IL-17A is consistent with previous findings from studies modeling MIA using IL-6 in human dorsal forebrain organoids^21^. To further confirm this, we treated neurons differentiated from iPSC-derived neural progenitor cells (NPCs) with IL-17A and performed Ca^2+^ imaging. IL-17A-treated neurons exhibited increased spontaneous neuronal activity, further supporting enhanced maturation (Extended Data Fig. 4a–c). Premature cortical folding, increased cortical thickness and accelerated neuronal maturation mirror clinical observations in some individuals with ASD, including macrocephaly^22,23^, an increased number of prefrontal cortical neurons^24^ and enhanced cortical folding^25^.

### Enhanced neurogenesis by IL-17A

**Fig. 3:**
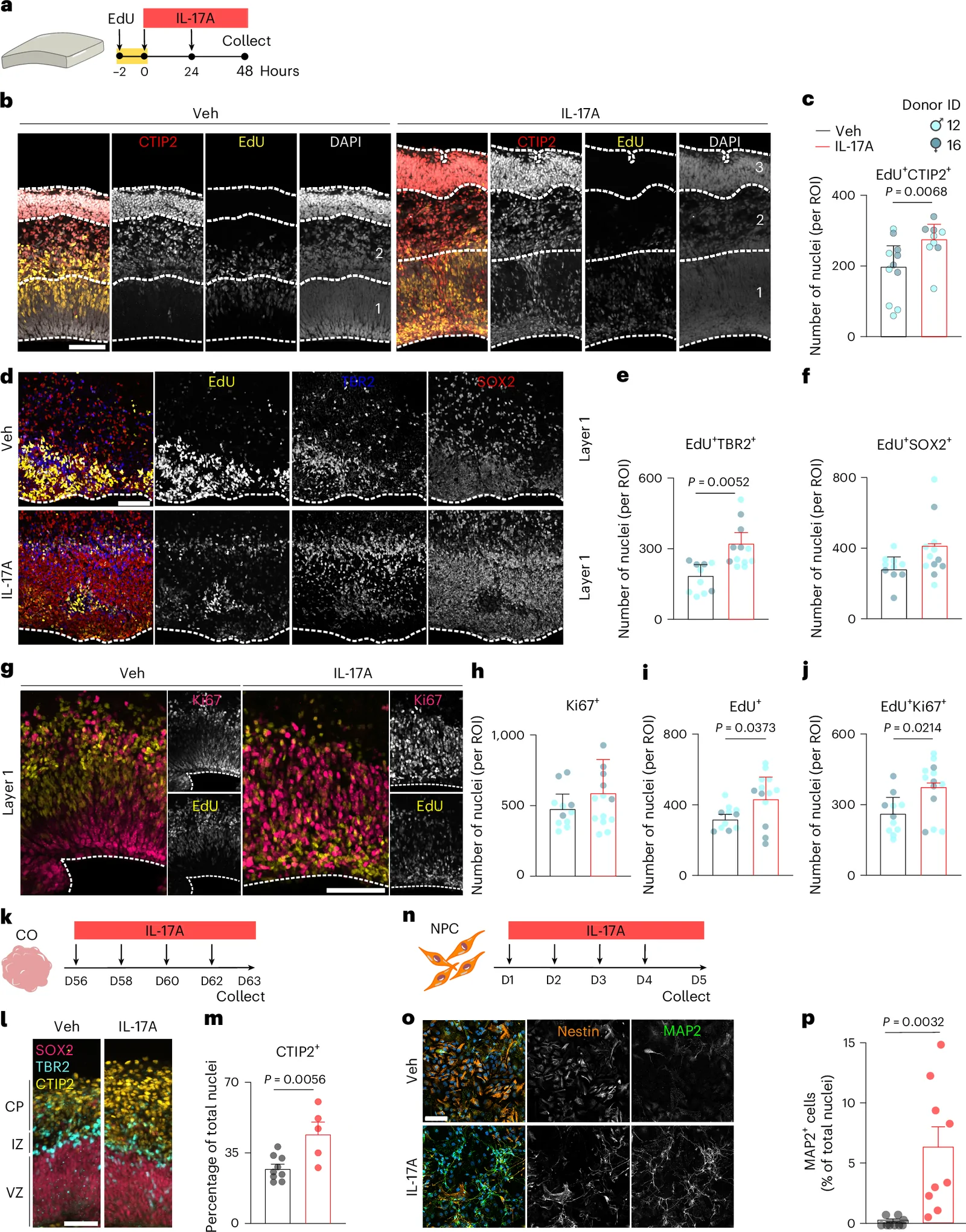
IL-17A affects the proliferation of NSCs and enhances neurogenesis. **a**, Schematic illustration of the experimental paradigm for IL-17A and EdU administration to cerebroids. **b**, Representative fluorescence images of EdU^+^CTIP2^+^ neurons in vehicle- and IL-17A-treated cerebroids. **c**, Quantification of EdU^+^CTIP2^+^ neurons in vehicle- and IL-17A-treated cerebroids. Two-way ANOVA (two-sided). *n* = 6 cerebroids from 2 donors in each group. **d**, Representative fluorescence images showing EdU incorporation in NSCs (SOX2^+^) and IPCs (TBR2^+^) in vehicle- and IL-17A-treated cerebroids. **e**,**f**, Quantification of EdU^+^TBR2^+^ IPCs (**e**) and EdU^+^SOX2^+^ NSCs (**f**) in vehicle- and IL-17A-treated cerebroids. Two-way ANOVA (two-sided). *n* = 6 cerebroids from 2 donors in each group. **g**, Representative fluorescence images showing EdU incorporation and cell cycle marker Ki67 in vehicle- and IL-17A-treated cerebroids. **h**–**j**, Quantification of the number of Ki67^+^ cells (**h**), EdU^+^ cells (**i**) and EdU/Ki67 double-positive cells (**j**) in vehicle- and IL-17A-treated cerebroids. Two-way ANOVA (two-sided). *n* = 6 cerebroids from 2 donors in each group. **k**, Schematic illustration of the experimental paradigm for IL-17A treatment of cortical organoids (CO) generated from human iPSCs. **l**, Representative fluorescence image of cortical organoids stained for SOX2 (NSCs), TBR2 (IPCs) and CTIP2 (neurons) after IL-17A treatment. **m**, Quantification of CTIP2^+^ cells. Unpaired *t*-test (two-sided). *n* = 7 (vehicle) and *n* = 5 (IL-17A) cortical organoids. **n**, Schematic illustration of the experimental paradigm for IL-17A treatment of iPSC-derived NPCs. **o**, Representative fluorescence images of Nestin^+^ (NPCs) and MAP2^+^ (neurons) in vehicle- and IL-17A-treated NPCs. **p**, Quantification of MAP2^+^ cells in vehicle- and IL-17A-treated NPCs. Unpaired *t*-test (two-sided). *n* = 9 replicates from three independent experiments in each group. For **c**, **e**, **f**, **h**, **i**, **j**, **m** and **p**, data are represented as mean ± s.e.m. Scale bar: 100 μm. Source data

To identify the cellular mechanisms underlying abnormal cortical development, we examined the acute impact of IL-17A on NSCs. To determine whether the increased number of CTIP2^+^ neurons in IL-17A-treated cerebroids was due to elevated nascent neurogenesis, we conducted an EdU pulse–chase experiment. Following a 2-h pulse and 48-h chase, we observed a higher number of EdU^+^CTIP2^+^ neurons in IL-17A-treated cerebroids, suggesting that IL-17A increases nascent neurogenesis (Fig. 3a–c). This phenotype was driven by an increased generation of TBR2^+^ IPCs, as we observed a higher number of EdU^+^TBR2^+^ cells in IL-17A-treated cerebroids (Fig. 3d,e). The prolonged course of neurogenesis is a key factor in cortical expansion in humans^26^, and premature differentiation of neural progenitors into neurons can deplete the progenitor pool, resulting in reduced neuronal numbers and often leading to a microcephaly phenotype^27^. Although we detected more EdU-labeled CTIP2^+^ neurons and TBR2^+^ IPCs in IL-17A-treated cerebroids, the number of EdU^+^SOX2^+^ NSCs remained comparable between the two groups (Fig. 3d,f), suggesting that enhanced neurogenesis was accompanied by increased proliferation and replenishment of the NSC population. Notably, while no difference in the number of Ki67^+^ proliferating NSCs was found between vehicle and IL-17A-treated groups, a greater number of EdU^+^ cells and EdU^+^Ki67^+^ cells were observed in IL-17A-treated cerebroids, indicating that a higher number of EdU^+^ cells re-entered the cell cycle. (Fig. 3g–j). To further support the observation of increased neurogenesis induced by IL-17A, iPSC-derived cortical organoids were treated with IL-17A. Consistent with findings in cerebroids, the number of CTIP2^+^ neurons was increased in the IL-17A-treated organoids (Fig. 3k–m). However, we did not observe a cortical folding phenotype in IL-17A-treated organoids, suggesting that ECM components and cell types absent in organoids may be crucial in mediating this phenotype. Additionally, IL-17A treatment of iPSC-derived NPCs resulted in accelerated neurogenesis even in the absence of neuronal differentiation media, as shown by an increased number of MAP2^+^ cells in the IL-17A-treated group (Fig. 3n–p). These findings from our organoid and NPC experiments suggest that the increased neurogenesis induced by IL-17A is due to a direct effect on NSCs, rather than being mediated by microglia-driven immune responses. This contrasts with recent findings in mice, where transcriptomic changes in both neuronal and non-neuronal cells in response to MIA were shown to require microglia^28^.

### Remodeling of ECM induced by IL-17A

**Fig. 4:**
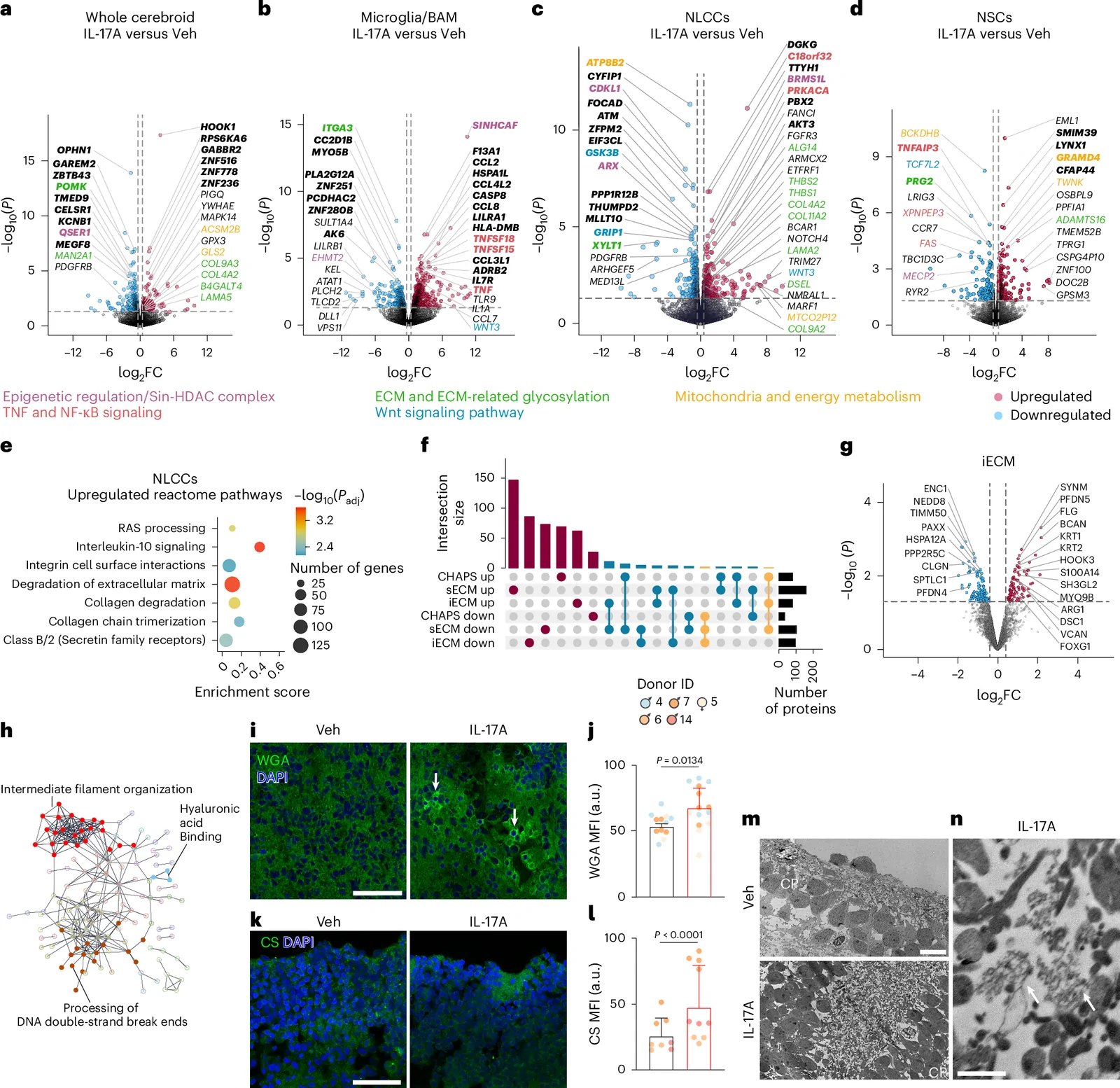
Transcriptional and proteomic alteration by IL-17A. **a**–**d**, Volcano plots of DEGs with a log_2_FC of 0.4 or greater in IL-17A- versus vehicle-treated cerebroids for bulk tissue (**a**), microglia (**b**), NLCCs (**c**) and NSCs (**d**). Red denotes upregulated and blue denotes downregulated genes (*n* = 3 donors). Differential expression was assessed using DESeq2 with a two-sided Wald test, and *P* values were adjusted for multiple comparisons using the Benjamini–Hochberg method. Genes with FDR-adjusted *P* < 0.05 are labeled in bold. Whole cerebroid RNA-seq was performed on donors 9, 11 and 12; RNA-seq on isolated cell types was performed on donors 5, 6 and 7 (see Supplementary Table 1 for donor information). **e**, Bubble plot of enriched Reactome pathways from protein sub-network-based gene set enrichment analysis, showing upregulated genes in NLCCs after IL-17A treatment. Bubble color indicates FDR-adjusted *P* value (Benjamini–Hochberg method), and size reflects the number of associated genes. **f**, UpSet plot showing the number of differentially abundant proteins across three fractions (CHAPS, sECM, iECM) in IL-17A- versus vehicle-treated cerebroids (*n* = 3 donors; donor IDs: 9, 11 and 12). **g**, Volcano plot of differentially abundant proteins (log_2_FC ≥ 0.4) in IL-17A- versus vehicle-treated cerebroids in the iECM fractions. Differential abundance was assessed using limma (moderated *t*-test, two-sided), with *P* values adjusted for multiple comparisons using the Benjamini–Hochberg method. No proteins met the FDR-adjusted significance threshold (FDR < 0.05). Proteins with nominal *P* < 0.05 are highlighted in red (upregulated) and blue (downregulated). **h**, Protein–protein interaction network of differentially abundant proteins in the iECM fraction, visualized using STRING clustering. Colors represent distinct sub-networks generated by *k*-means clustering. **i**, Representative immunofluorescence images of WGA staining in vehicle- and IL-17A-treated cerebroids. Arrows indicate stronger WGA staining in IL-17A-treated cerebroids. **j**, Quantification of the MFI of WGA in layer 2 and layer 3 of vehicle- and IL-17A-treated cerebroids. Two-way ANOVA (two-sided). *n* = 9 cerebroids from 3 donors in each group. **k**, Representative fluorescence images of chondroitin sulfate staining, showing enhanced chondroitin sulfate signal intensity at the CP folding site in IL-17A-treated cerebroids. **l**, Quantification of the MFI of chondroitin sulfate in layer 3 of vehicle- and IL-17A-treated cerebroids. Two-way ANOVA (two-sided). *n* = 8 (vehicle) and *n* = 10 (IL-17A) cerebroids from 3 donors. **m**,**n**, TEM images of CPregions showing areas of folding (asterisk) with abundant ECM in IL-17A-treated cerebroids (**m**). Higher magnification of glycosaminoglycan-containing proteoglycans (indicated by arrows) in the ECM (**n**). For **j** and **l**, data are represented as mean ± s.e.m. Scale bars: 50 μm (**i**, **k**), 5 μm (**m**) and 500 nm (**n**). CS, chondroitin sulfate; FC, fold change. Source data

**Extended Data Fig. 5:**
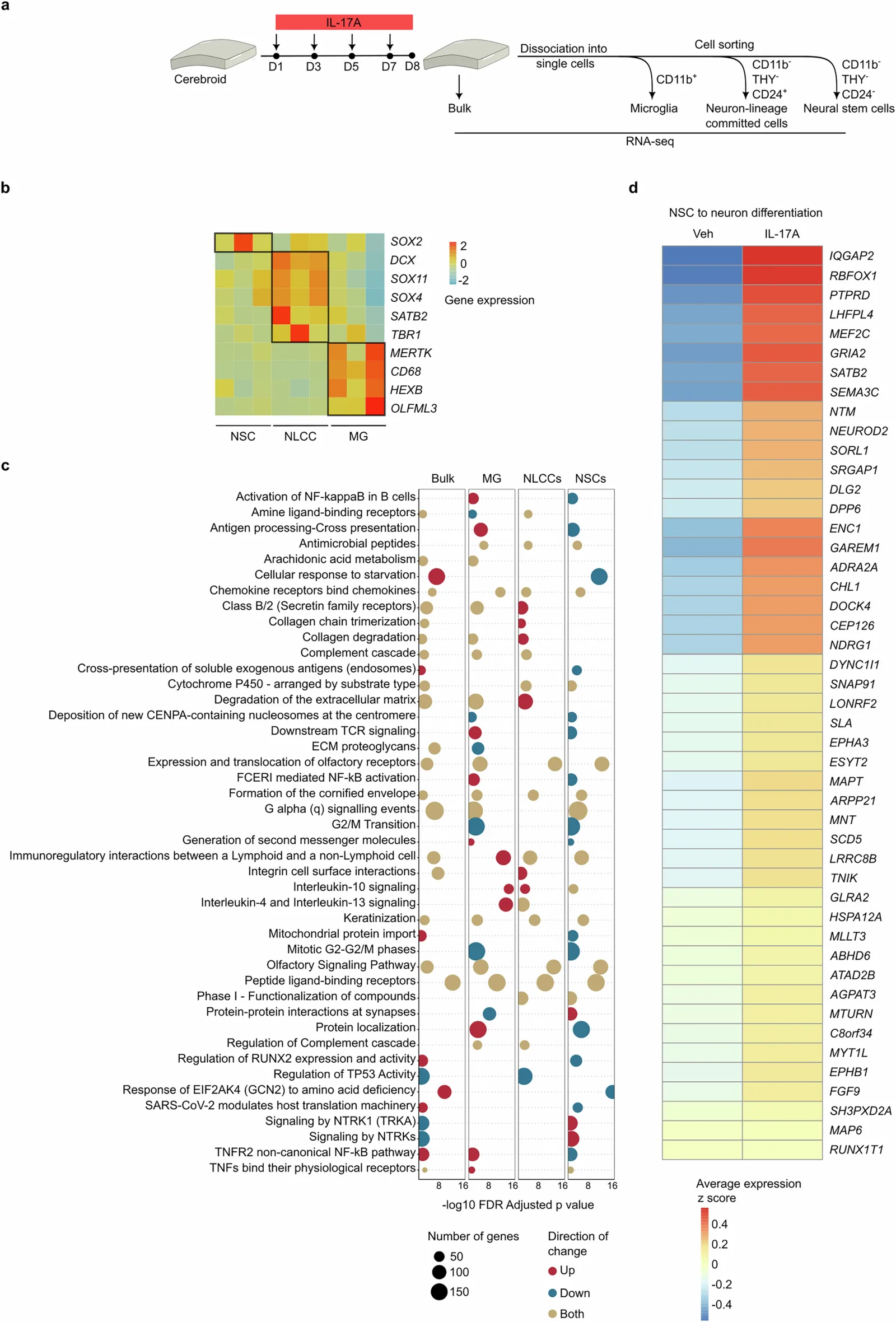
Marker genes for sorted cell populations and functional enrichment analysis of transcriptomic data. **a**, Schematic of the experimental design for RNA sequencing in bulk cerebroid tissue, microglia (CD11b^+^), neuron-lineage committed cells (NLCCs) (CD11b^−^, THY^−^ and CD24^+^), and NSCs (CD11b^−^, THY^−^ and CD24^−^). **b**, Heatmap showing scaled expression of selected marker genes in NSCs (*SOX2*), neuron-lineage committed cells (NLCCs, *DCX*, *SOX11*, *SOX4*, *SATB2*, *TBR1*), and microglia (MG, *MERTK*, *CD68*, *HEXB*, *OLFML3*) isolated from Day 8 cerebroids (n = 3 donors). The heatmap highlights the distinct expression profiles of these marker genes across the sorted cell populations. **c**, Bubble plot illustrating enriched Reactome pathways identified in two or more RNA-seq datasets (whole cerebroid, MG, NLCCs, and NSCs). Pathway enrichment was determined using protein network-based gene set enrichment analysis via STRING, with genes ranked by Log2FC. Enrichment P values were calculated using a two-sided hypergeometric test and adjusted for multiple comparisons using the Benjamini–Hochberg method. Only pathways enriched in at least two datasets and involving ≥50 genes are shown. Each column represents a transcriptomic dataset, with the position of the bubble along the x-axis indicating the -log10 of the FDR-adjusted p-value. Bubble size corresponds to the number of genes associated with each pathway, while colour denotes the direction of gene expression changes. **d**, Heatmap of DESeq2-normalized and VST-transformed read counts from NSCs isolated from vehicle- and IL-17A-treated day 8 cerebroids from 3 donors. Z-scores were calculated for gene expression and mean z-scores across 3 donors in each group are plotted. Heatmap shows higher expression of genes involved in neurogenesis from NSCs. Source data

**Extended Data Fig. 6:**
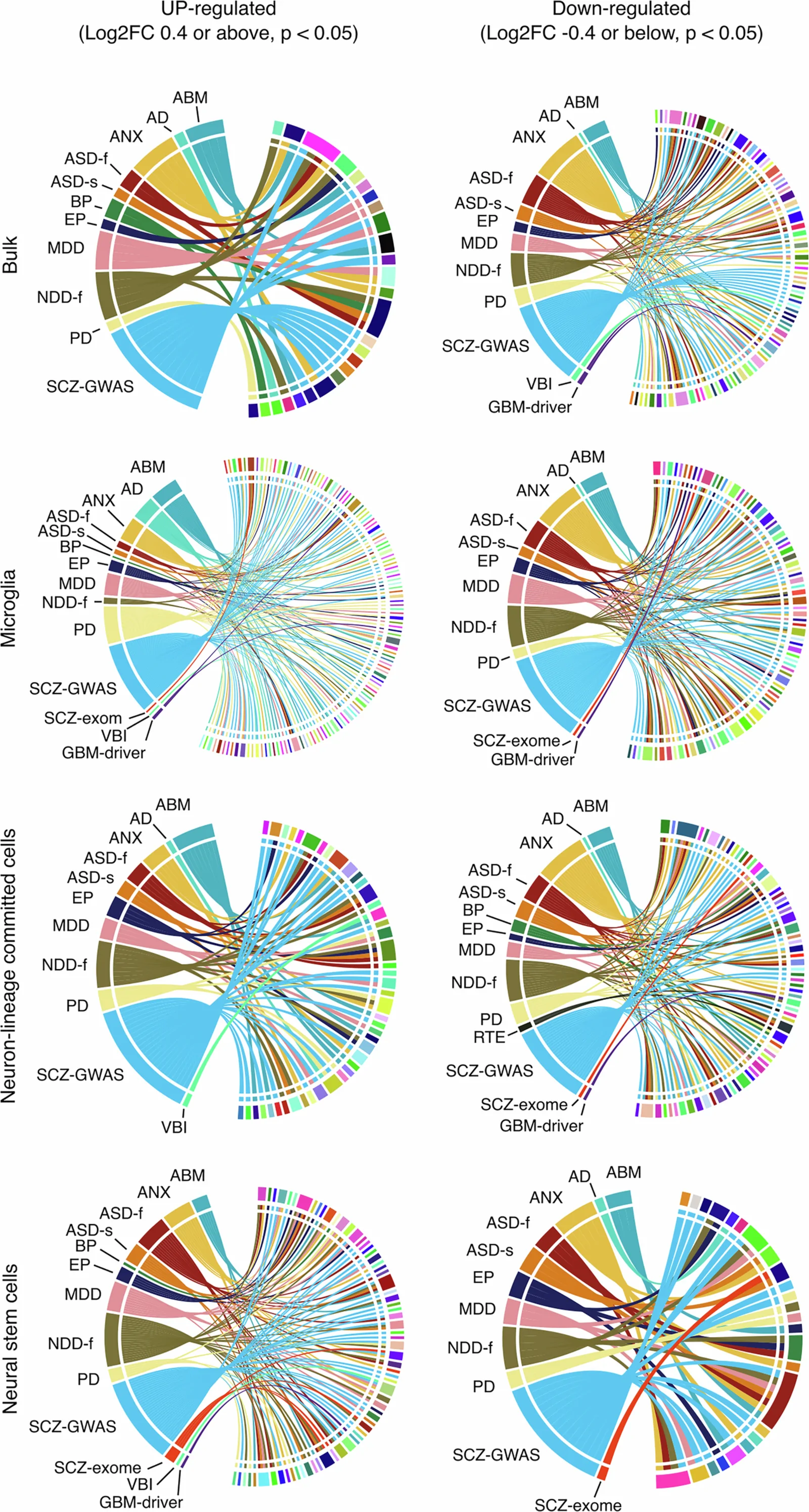
Differentially expressed genes in IL-17A-treated cerebroids are linked to neurological disorders. Chord plots showing the association of a subset of differentially expressed genes (DEGs) from IL-17A-treated cerebroids (whole cerebroids and subpopulations) with various neurological disorders. The list of genes is taken from a recent study^93^. Disorders are listed on the left, with DEGs on the right. Abbreviations: ABM (abnormal brain morphology), AD (Alzheimer’s disease), ANX (anxiety disorder), ASD-f (Autism spectrum disorders, genes selected from Fu et al.), ASD-s (Autism spectrum disorders, genes selected from Satterstrom et al.), BP (bipolar disorder), EP (epilepsy), MDD (major depressive disorder), NDD-f (neurodevelopmental disorders, genes selected from Fu et al.), PD (Parkinson’s disease), RTE (response to trauma exposure), SCZ-GWAS (schizophrenia genes identified through genome-wide association studies), SCZ-exom (schizophrenia genes identified through exome sequencing), VBI (vascular brain injury), GBM-driver (glioblastoma multiforme driver genes).

**Extended Data Fig. 7:**
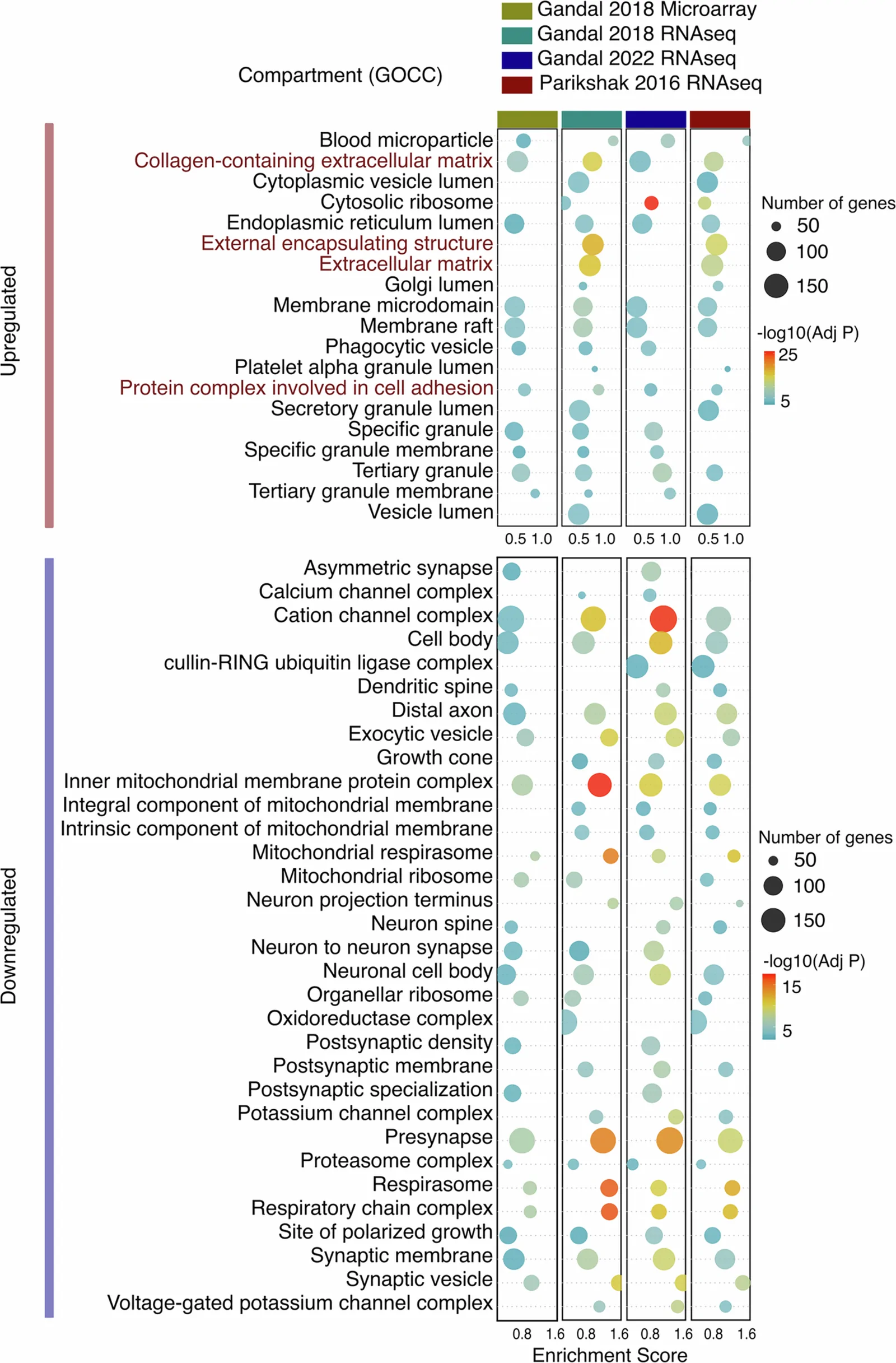
Meta-analysis of bulk transcriptomic data from post-mortem brains of individuals with ASD. Bubble plot displaying gene ontology cellular compartment (GOCC) enrichment for differentially regulated genes from bulk transcriptomic meta-analyses of the frontal and whole cortex of individuals with autism spectrum disorder (ASD). The analysis includes data from a meta-analysis of microarray studies and three RNA-seq studies. Enrichment analysis was performed using the STRING database, with genes ranked by Log2FC for each dataset separately. The X-axis represents the enrichment score for each term, as calculated by STRING, while columns represent individual transcriptomic datasets. Bubble size corresponds to the number of genes associated with each term, and colour reflects the -log10 of the FDR-adjusted P-value. Only terms shared across two or more datasets, involving ≥50 genes, are shown. Enriched terms for both upregulated and downregulated genes are shown. ECM related terms are highlighted in red. Source data

To uncover the molecular mechanisms underlying abnormal cortical development, we performed bulk RNA-seq on whole cerebroids, as well as on three isolated cell populations: microglia/BAMs, neuron-lineage-committed cells (NLCCs) and NSCs (Extended Data Fig. 5a)^29^. The cellular identities of these isolated populations were confirmed by expression levels of marker genes (Extended Data Fig. 5b). When comparing differentially expressed genes (DEGs) between vehicle-treated control and IL-17A-treated cerebroids, we identified five common categories of DEGs, including pathways related to epigenetic regulation/Sin-HDAC complex, TNF/NF-κB signaling, ECM and ECM-related glycosylation, energy metabolism and WNT signaling (Fig. 4a–d). While each population exhibited distinct Reactome pathway alterations, ECM-related pathways were consistently enriched across all cell types analyzed (Fig. 4a–d, Extended Data Fig. 5c and Supplementary Tables 2–9). Specifically, in NLCCs, IL-17A upregulated genes involved in ECM remodeling processes, including *THBS2*, *THBS1*, *COL4A2*, *COL11A2*, *LAMA2*, *DSEL* and *COL9A2*, which are associated with integrin cell-surface interactions, ECM degradation, collagen degradation and collagen chain trimerization in Reactome enrichment analysis (Fig. 4e). Mounting evidence shows that mitochondrial dynamics substantially influence cellular metabolism and shape the behavior of NSCs during brain development^30^. Notably, mitochondria-related metabolic pathways were uniquely enriched in the Reactome pathway analysis of NSCs (Fig. 4d and Supplementary Table 9), suggesting that altered mitochondria dynamics induced by IL-17A may contribute to the increased neurogenesis. This observation is consistent with the upregulated expression of neuronal differentiation-associated genes in NSCs from IL-17A-treated cerebroids (Extended Data Fig. 5d). Moreover, many DEGs in IL-17A-treated cerebroids were associated with neurodevelopmental and neurological disorders (Extended Data Fig. 6), suggesting that IL-17A may dysregulate shared pathways involved in these conditions. We further conducted a meta-analysis of transcriptomic data from post-mortem brains of individuals with ASD (Extended Data Fig. 7 and Supplementary Table 10)^31–33^. Enrichment analysis revealed that many upregulated genes are linked to ECM components, reinforcing the significance of the ECM in the pathogenesis of NDDs.

**Extended Data Fig. 8:**
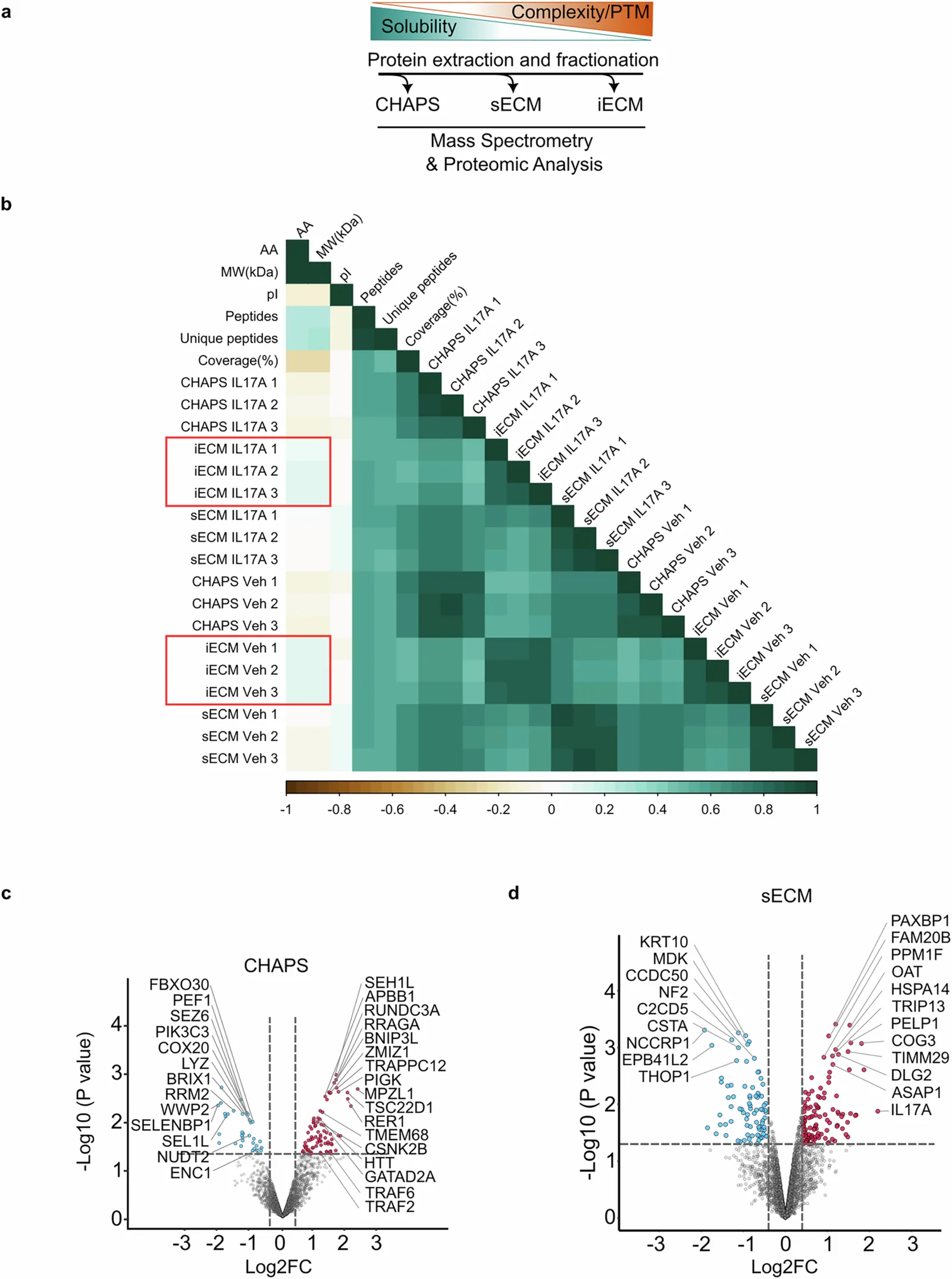
Proteomic analysis of IL-17A-treated cerebroids. **a**, Schematic of protein extraction for mass spectrometry. Cellular proteins (CHAPS fraction), soluble extracellular matrix (sECM), and insoluble extracellular matrix (iECM) fractions were isolated and analysed separately. **b**, Correlogram showing the relationships between protein properties and their abundance across different fractions from day 8 vehicle- and IL-17A-treated cerebroids. Colours indicate the strength and direction of correlations. In the iECM fraction, protein abundance positively correlates with protein complexity, as represented by the number of amino acids (AA) and molecular weight (MW, kDa). Conversely, negative correlations are observed between protein complexity (AA and MW) and the sECM and CHAPS fractions, suggesting enrichment of larger, more complex proteins in the iECM fraction. pI: isoelectric point. **c,d**, Volcano plot depicting differentially abundant proteins in the CHAPS (**c**) and sECM fraction (**d**) between IL-17A- and vehicle-treated day 8 cerebroids.

**Extended Data Fig. 9:**
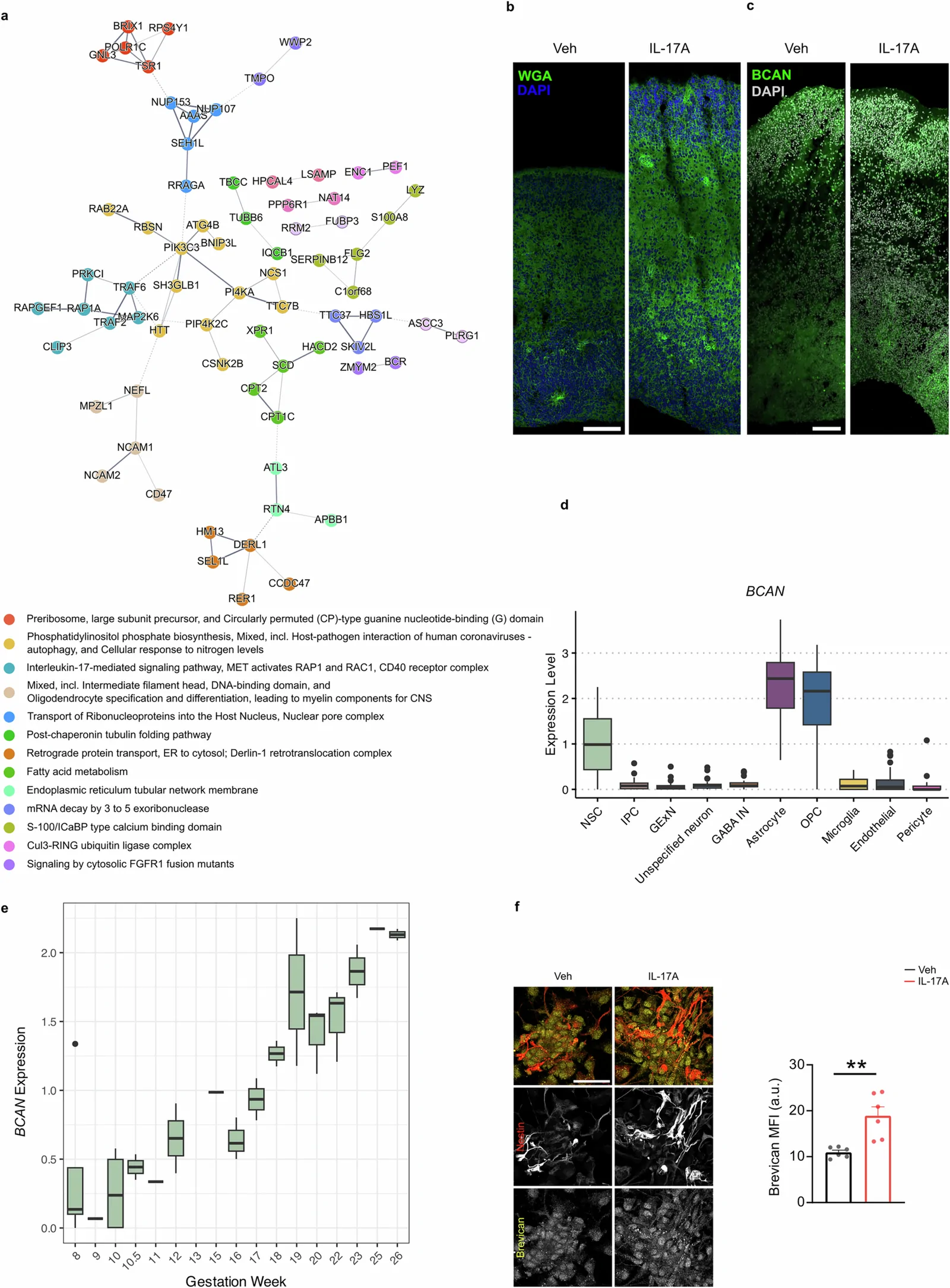
Protein-protein interaction network of differentially abundant proteins and validation of ECM alterations in IL-17A-treated cerebroids. **a**, Protein-protein interaction network of differentially abundant proteins from the CHAPS fraction of IL-17A-treated versus vehicle-treated day 8 cerebroids. The network was constructed using the STRING database, where each node represents a protein, and each line indicates a protein-protein interactions. The thickness of the lines corresponds to the confidence level of the interaction evidence. Unconnected nodes are omitted. K-means clustering was applied to the network, with each color representing a distinct protein cluster associated with an enriched biological term. **b**, Representative fluorescence images of Day 8 vehicle- and IL-17A-treated cerebroids stained with Alexa fluor conjugated WGA and DAPI. WGA binds to N-acetylglucosamine and N-acetylneuraminic acid (sialic acid) residues and labels glycocalyx and glycoprotein containing ECM. **c**, Representative fluorescence images of vehicle- and IL-17A-treated cerebroids stained for the ECM component BCAN. Scale bar, 100 μm. **d**, A boxplot of expression levels of *BCAN* by various cell types in the developing human cortex. *BCAN* is highly expressed by NSCs, Astrocytes and oligodendrocyte progenitors (OPCs). **e**, Temporal expression pattern of *BCAN* in NSCs from GW 8 to 26 of developing human cortex. A temporal increase in *BCAN* expression is observed in NSCs across fetal brain development. In all box plots in (**d**, **e**), the center line represents the median, the box bounds correspond to the 25th and 75th percentiles, whiskers extend to the minimum and maximum values, and any points beyond the whiskers are defined as outliers. Source: Gim et al.^45^. n = 37 (NSC), n = 33 (IPC), n = 36 (GExN), n = 37 (unspecified neuron), n = 34 (GABA IN), n = 24 (Astrocyte), n = 31 (OPC), n = 32 (microglia), n = 30 (Endothelial), n = 30 (Pericytes) donors across 6 independent studies. **f**, Representative images and quantification of BCAN expression by human iPSC-derived NPCs after 48 h treatment with the vehicle- or IL-17A. Data are represented as mean ± s.e.m.; Unpaired t-test (two-sided): t(10) = 3.966, P = 0.0027; means 10.9 (vehicle) and 18.9 (IL-17A); difference 8.0 (95% CI 3.5–12.5), η^2^ = 0.611. **p value < 0.01. (n = 6 from 3 independent experiments in each group). Source data

To examine the ECM alterations induced by IL-17A, we performed mass spectrometry and proteomic analysis of three different protein fractions based on the solubility and complexity of proteins: the CHAPS fraction, containing cellular proteins, the soluble ECM (sECM) fraction and the insoluble ECM (iECM) fraction (Extended Data Fig. 8a,b). We observed more than 400 differentially abundant sECM and iECM proteins in IL-17A-treated cerebroids. (Fig. 4f,g, Extended Data Fig. 8c,d and Supplementary Tables 11–13). Protein–protein interaction network analysis of differentially abundant proteins in the CHAPS fraction of IL-17A-treated cerebroids revealed host–pathogen interaction of virus- and IL-17A-mediated signaling pathways, highlighting the activation of immune responses by IL-17A (Extended Data Fig. 9a). In the iECM fraction of IL-17A-treated cerebroids, two key chondroitin sulfate proteoglycans, BCAN and versican (VCAN), were upregulated (Fig. 4g). BCAN is a major proteoglycan in the brain’s ECM, playing critical roles in neural development, synaptic plasticity and neuronal stability^34–37^. VCAN contributes to the structural integrity of the developing ECM and is involved in cell proliferation, migration and synapse formation^38–41^. Both BCAN and VCAN bind to hyaluronic acid, a key ECM component that regulates cortical folding, cell migration, proliferation, axon guidance and synapse formation^20,42^. Hyaluronic acid binding was also highlighted in the protein–protein interaction network in the iECM fraction of IL-17A-treated cerebroids, along with intermediate filament organization and processing of DNA double-strand break ends (Fig. 4h).

The altered proteoglycan expression was visualized using wheat germ agglutinin (WGA), showing overall upregulation and increased perineuronal net immunoreactivity in IL-17A-treated cerebroids (Fig. 4i and Extended Data Fig. 9b). Additionally, chondroitin sulfate immunoreactivity was elevated at the sites of cortical folding in IL-17A-treated cerebroids (Fig. 4j), aligning with evidence implicating the ECM in cortical folding^43,44^. TEM further confirmed the presence of increased glycosaminoglycan-containing proteoglycans at the folding sites at the ultrastructural level (Fig. 4k,l). These findings suggest a strong association between altered ECM composition and IL-17A-induced cortical folding. We also observed that expression of BCAN was heightened in ventricular zones of IL-17A-treated cerebroids (Extended Data Fig. 9c). To identify the cell type(s) contributing to increased BCAN expression, we analyzed single-cell RNA-seq meta-data from the developing human cortex, comprising 37 donors between gestational weeks 8 and 26 across six independent studies^45^. While astrocytes and oligodendrocyte precursor cells show the highest BCAN expression, NSCs also express BCAN at notable levels (Extended Data Fig. 9d). Given the limited number of astrocytes and oligodendrocyte precursor cells at this developmental stage, NSCs are likely the primary source of BCAN. BCAN expression also increases progressively during development (Extended Data Fig. 9e). Accordingly, enhanced BCAN expression following IL-17A treatment is consistent with other accelerated maturation phenotypes, including increased SATB2^+^ and NeuN^+^ neurons in cerebroids (Fig. 2j–n) and enhanced spontaneous neuronal activity in iPSC-derived neurons (Extended Data Fig. 4). Elevated BCAN expression in NSCs was further confirmed in human iPSC-derived NPCs (Extended Data Fig. 9f). These alterations in ECM components are particularly noteworthy considering recent findings showing increased BCAN and VCAN expression in post-mortem brains of children with ASD^46^.

### Activation of NF-κB signaling by IL-17A in NSCs

**Fig. 5:**
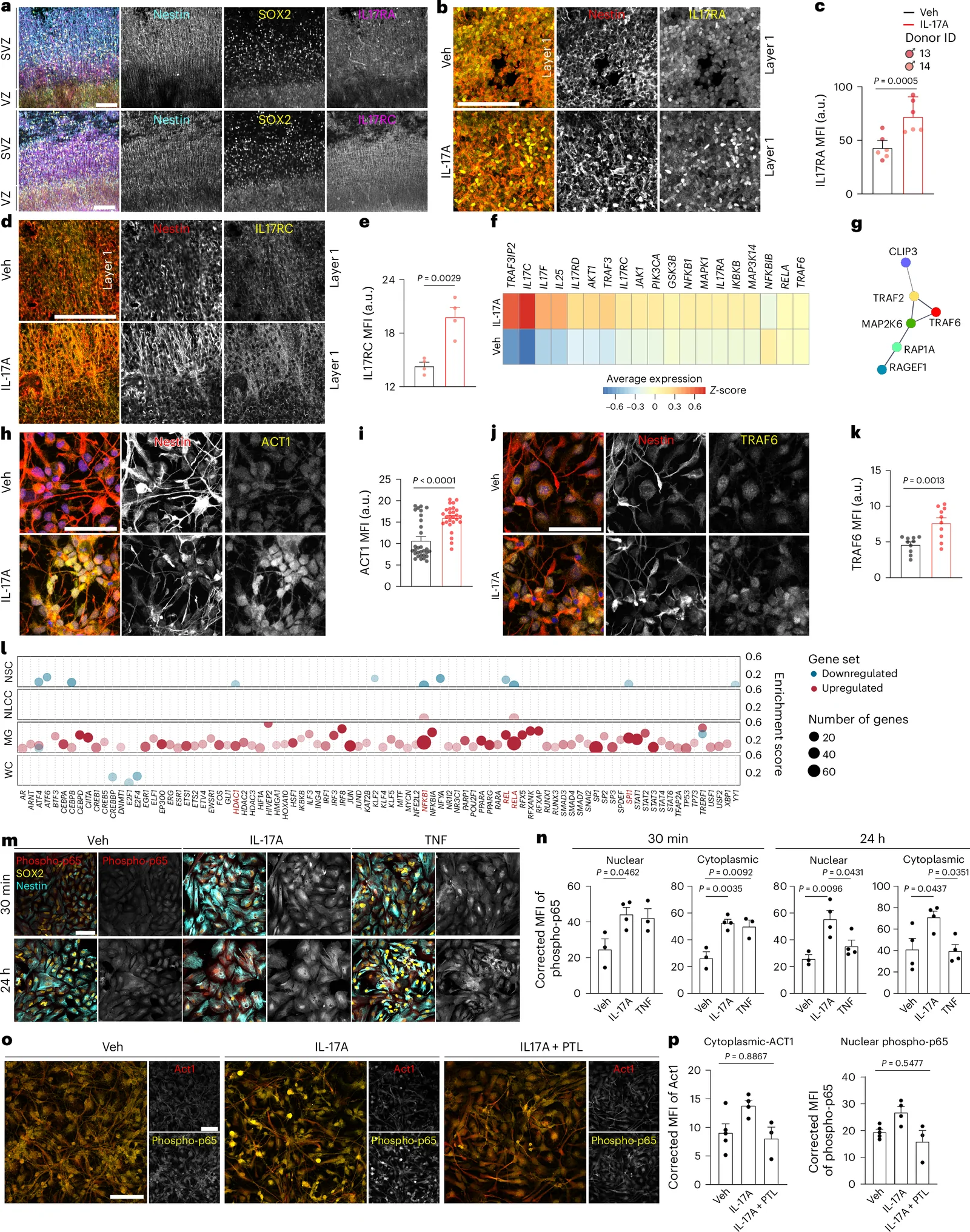
Expression of IL-17A receptors by NSCs and activation of IL-17A and NF-κB signaling pathways upon IL-17A treatment. **a**, Representative maximum intensity projection images of gestational week 12 fetal DLPFC showing IL-17A receptor expression: IL17RA (top) and IL17RC (bottom). **b**,**c**, Representative images (**b**) and quantification (**c**) of IL17RA expression in Nestin^+^ NSCs in layer 1 of day 8 vehicle- and IL-17A-treated cerebroids. Two-way ANOVA (two-sided). *n* = 6 cerebroids from 2 donors in each group. **d**,**e**, Representative images (**d**) and quantification (**e**) of IL17RC expression in NSCs in layer 1 of day 8 vehicle- and IL-17A-treated cerebroids. Unpaired *t*-test (two-sided). *n* = 4 cerebroids from 1 donor in each group. **f**, Heatmap of DESeq2-normalized, VST-transformed mean expression of IL-17 pathway genes in NSCs from day 8 vehicle- and IL-17A-treated cerebroids. Average *Z*-scores across 3 donors are shown. **g**, STRING network of differentially abundant proteins from the cellular fraction of day 8 cerebroids, highlighting IL-17A and NF-κB pathway components. **h**,**i**, Representative images (**h**) and quantification (**i**) of ACT1 expression in iPSC-derived NPCs after 48 h of treatment with vehicle or IL-17A. Unpaired *t*-test (two-sided). *n* = 30 (vehicle) and *n* = 27 (IL-17A) high-magnification images analyzed from three independent experiments. **j**,**k**, Representative images (**j**) and quantification (**k**) of TRAF6 expression in 2D cultured NPCs after 48 h of treatment with vehicle or IL-17A. Unpaired *t*-test (two-sided). *n* = 10 high-magnification images analyzed across 3 independent experiments in each group. **l**, Bubble plot of significant human transcription factors from the TRRUST database regulating DEGs in IL-17A-treated whole cerebroids (WC), microglia (MG), NLCCs and NSCs. Bubble color indicates direction of change, and size represents the number of regulated DEGs (FDR-adjusted *P* < 0.05). Analysis was performed with Enrichr. **m**, Representative images of human iPSC-derived NPCs treated with vehicle, IL-17A or TNF for 30 min or 24 h and stained for phosphorylated p65. **n**, Quantification of nuclear and cytoplasmic phosphorylated p65 fluorescence intensity at 30 min and 24 h after treatment with vehicle, IL-17A or TNF. One-way ANOVA with Tukey’s multiple-comparisons test. *n* = 3 (Veh and TNF) and *n* = 4 (IL-17A) at 30 min; *n* = 4 in each group at 24 h. *n* represents independent experiments. **o**, Representative images of iPSC-derived NPCs treated for 30 min with vehicle, IL-17A or IL-17A plus PTL. **p**, Quantification of cytoplasmic ACT1 fluorescence intensity and nuclear phosphorylated p65 after 30 min of treatment. One-way ANOVA with Tukey’s multiple-comparisons test. *n* = 4 (Veh and IL-17A) and *n* = 3 (IL-17A + PTL). *n* represents independent experiments. For **c**, **e**, **i**, **k**, **n** and **p**, data are represented as mean ± s.e.m. Scale bars: 100 µm (**a**) and 50 µm (**b**, **d**, **h**, **j**, **m**, **o**). SVZ, subventricular zone. Source data

**Extended Data Fig. 10:**
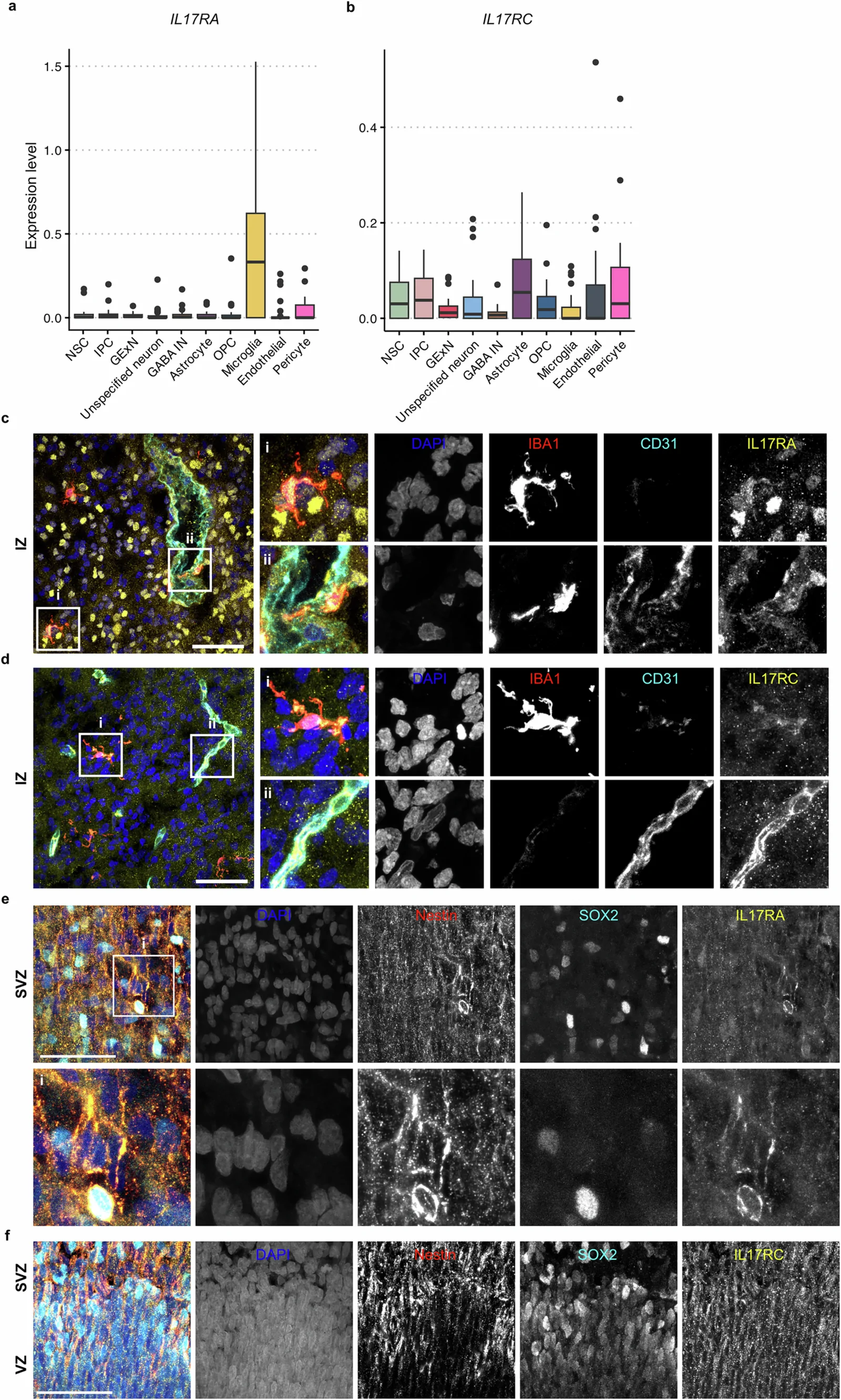
Expression of IL-17RA and IL-17RC. **a**, **b**, Boxplots showing the expression levels of IL17RA (a) and IL17RC (b) based on single-cell RNA sequencing meta-analysis of the developing human cortex spanning gestational weeks (GW) 8–26 from six independent studies. IL17RA is most highly expressed in microglia and pericytes, whereas IL17RC shows more widespread expression across multiple cell types, including NSCs at lower levels. Notably, IL17RC expression in some cell types, including NSCs, is higher than in microglia. In all boxplots, the center line represents the median, box bounds indicate the 25th and 75th percentiles, whiskers extend to the minimum and maximum values, and points beyond the whiskers are shown as outliers. Meta-analysis source: Gim et al.^45^. n = 37 (NSC), n = 33 (IPC), n = 36 (GExN), n = 37 (unspecified neuron), n = 34 (GABA IN), n = 24 (Astrocyte), n = 31 (OPC), n = 32 (microglia), n = 30 (Endothelial), n = 30 (Pericytes) donors across 6 independent studies. **c**, **d**, Maximum intensity projection fluorescence images of the GW14 cortical IZ showing expression of IL17RA (**c**) and IL17RC (**d**) in IBA1^+^ microglia and CD31^+^ vascular endothelial cells. **e**, **f**, Maximum intensity projection images showing expression of IL17RA (**e**) and IL17RC (**f**) in SOX2^+^Nestin^+^ NSCs. In (e), higher-magnification image shows IL17RA localization in the soma and Nestin^+^ processes of NSCs. Note that IL17RA immunoreactivity was predominantly localized to the soma, while IL17RC was mainly observed along radial processes and the cell membrane. Scale bar, 50 µm.

To investigate the molecular pathways in NSCs that mediate abnormal cortical development under IL-17A-mediated MIA, we investigated whether NSCs directly respond to IL-17A and characterized the downstream signaling mechanisms. We first examined the expression of IL-17A receptors in NSCs. Analysis of the developing cortex at both transcriptomic and protein levels revealed expression of the IL-17A receptors IL17RA and IL17RC in NSCs (Extended Data Fig. 10a–f and Fig. 5a). Importantly, their expression levels were further upregulated following IL-17A exposure, suggesting that NSCs are not only competent to respond to IL-17A under baseline conditions but also increase receptor expression upon stimulation (Fig. 5b–e).

To assess the downstream signaling consequences of receptor engagement, we examined the expression of genes associated with IL-17A signaling in RNA-seq of NSCs isolated from IL-17A-treated cerebroids. This analysis revealed increased expression of genes in the IL-17 signaling pathway, including *TRAF3IP2*, which encodes ACT1, a critical adaptor protein that bridges IL-17 receptors to NF-κB activation (Fig. 5f). In parallel, we observed downregulation of *NFKBIB* (Fig. 5f) and *TNFAIP3* (Fig. 4d), negative regulators of NF-κB, consistent with a shift toward sustained inflammatory signaling. These transcriptional changes provide strong evidence that IL-17A promotes durable NF-κB activation in NSCs rather than a transient or self-limiting response. To further substantiate this conclusion, we employed complementary proteomic analysis. Proteomic profiling of the CHAPs fraction from IL-17A-treated cerebroids revealed differential abundance of several NF-κB-associated proteins in response to IL-17A, including direct activators such as TRAF6 and TRAF2^47^, and indirect activators such as MAP2K6, RAP1A, RAPGEF1 and CLIP3^48–50^ (Fig. 5g). Furthermore, we confirmed the upregulated expression of ACT1 and TRAF6 upon IL-17A treatment in NPCs using immunofluorescence (Fig. 5h–k). In addition, to identify key transcriptional regulators modulating the expression of DEGs from our RNA-seq analysis, we performed transcription factor enrichment analysis using Enrichr. Among the many transcriptional regulators, five, encoded by *HDAC1*, *NFKB1*, *REL*, *RELA* and *SPI1*, were found to be shared across at least two different cell types and four of them are associated with NF-κB signaling, confirming a clear NF-κB activation signature in IL-17A-treated cerebroids (Fig. 5l). These orthogonal approaches converge on the same conclusion that IL-17A activates NF-κB signaling in the developing human cortex.

We next sought to functionally validate these findings using human iPSC-derived NPCs. We confirmed that IL-17A robustly induces phosphorylation of the NF-κB subunit p65, accompanied by its nuclear translocation. Unlike TNF, used as a positive control for activation of NF-κB signaling, which elicited only a transient activation, IL-17A maintained nuclear p65 localization even after 24 h, highlighting the persistent nature of the response (Fig. 5m,n). Immunofluorescence experiments further showed that increased expression of phospho-p65 after IL-17A treatment was rescued by co-treatment with PTL, a pharmacological NF-κB inhibitor through its suppression of IκB kinase activity^47^ (Fig. 5o,p).

Together, these results provide multimodal evidence, spanning transcriptomics, proteomics and pharmacological functional assay, that IL-17A directly engages its receptors on NSCs and activates NF-κB signaling in a sustained manner. This sustained activation distinguishes IL-17A from other inflammatory cytokines such as TNF and points to a mechanism by which MIA may drive prolonged inflammatory states in the developing human cortex.

### Reversed abnormal cortical development by PTL

**Fig. 6:**
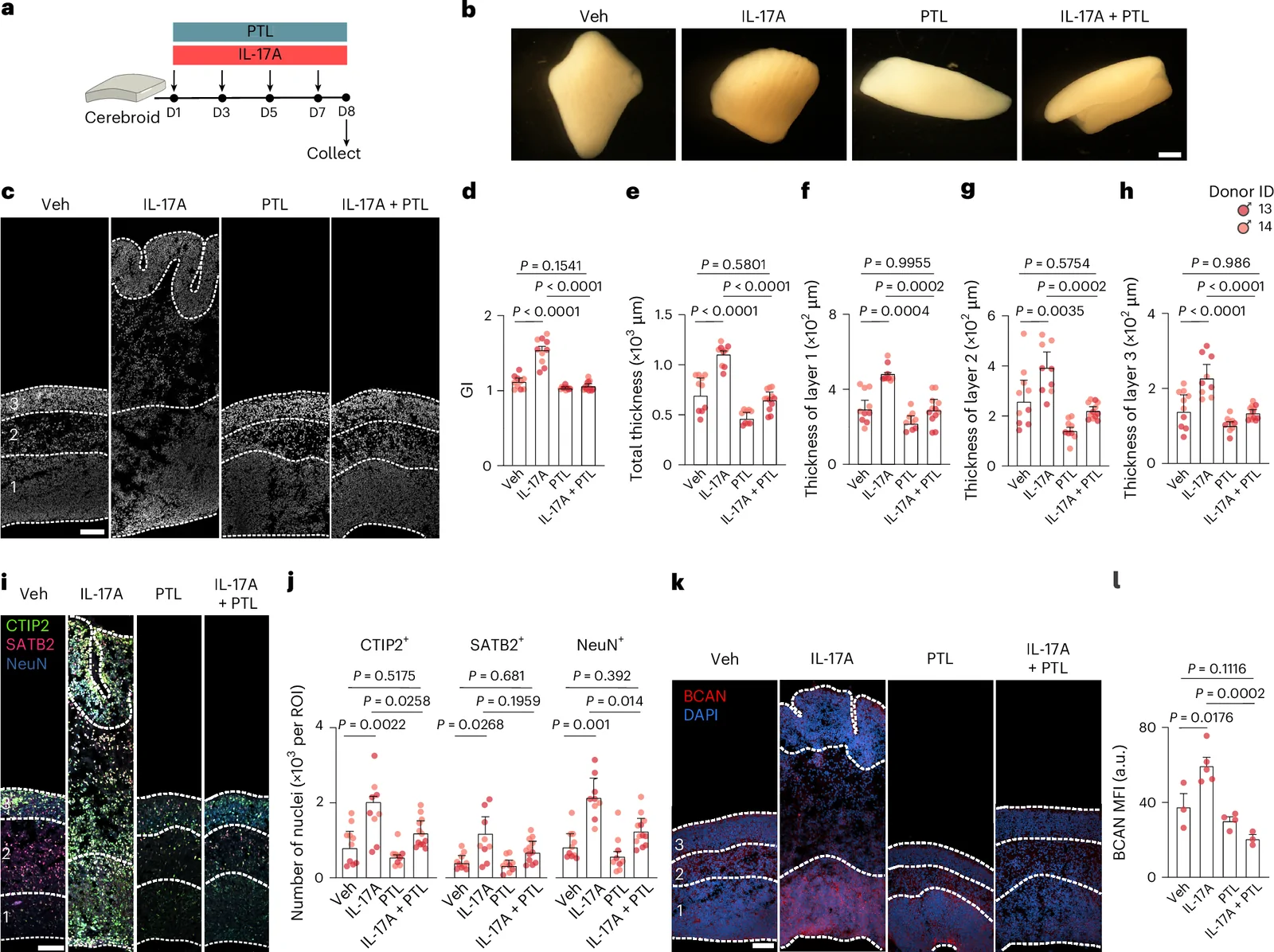
PTL reverses IL-17A-induced effects on cortical folding and neurogenesis in cerebroids. **a**, Schematic illustration of PTL and IL-17A administration to cerebroid cultures. **b**, Representative images of cerebroids treated with vehicle, IL-17A, PTL and IL-17A + PTL. **c**, DAPI-stained sections of cerebroids showing cortical architecture changes across treatment groups. Layer 1: VZ–SVZ; layer 2: IZ–SP; and layer 3: CP. **d**, Quantification of GI across treatment groups. Two-way ANOVA (two-sided) with Tukey’s multiple-comparisons test. *n* = 5 (Veh, IL-17A and PTL) and *n* = 6 (IL-17A + PTL) cerebroids from 2 donors. **e**–**h**, Quantification of total cortical thickness (**e**), as well as thickness of layer 1 (**f**), layer 2 (**g**) and layer 3 (**h**) in different treatment groups. **i**,**j**, Representative immunofluorescence images (**i**) and quantification of CTIP2- and SATB2-expressing neurons (**j**) in cerebroids across different treatment groups. Two-way ANOVA (two-sided) with Tukey’s multiple-comparisons test. *n* = 5 (Veh and PTL), *n* = 6 (IL-17A + PTL) and *n* = 7 (IL-17A) cerebroids from 2 donors **k**,**l**, Representative immunofluorescence images (**k**) and quantification (**l**) of BCAN across treatment groups. One-way ANOVA (two-sided) with Tukey’s multiple-comparisons test. *n* = 3 (Veh and IL-17A + PTL), *n* = 4 (PTL) and *n* = 5 (IL-17A) cerebroids from 1 donor. For **d**–**h**, **j** and **l**, data are presented as mean ± s.e.m. Scale bars: 1,000 μm (**b**) and 100 μm (**c**, **i**, **k**). Source data

To confirm the involvement of NF-κB signaling pathways in the abnormal cortical development phenotype, cerebroids were treated with PTL (Fig. 6a). While PTL alone did not induce any observable phenotype, it reversed the cortical folding and thickness abnormalities across different cortical layers in IL-17A-treated cerebroids (Fig. 6b–h). Furthermore, PTL treatment mitigated the excessive neurogenesis, as evidenced by a reduction in the number of CTIP2^+^, SATB2^+^ and NeuN^+^ neurons in IL-17A-treated cerebroids (Fig. 6i,j). Given the upregulation of BCAN in the ventricular zone of IL-17A-treated cerebroids (Extended Data Fig. 9c), we investigated whether PTL could reverse this effect as well. Following PTL treatment, BCAN expression in the ventricular zone was decreased, reaching levels similar to those observed in the vehicle group (Fig. 6k,l). These data demonstrate that the NF-κB pathway plays a crucial role in IL-17A-mediated abnormal cortical development during MIA.

## Discussion

In this study, we developed a robust high-throughput 3D ex vivo culture system, cerebroids, which faithfully preserve key components of cortical development such as cellular architecture, apical–basal polarity and essential immune, vascular and ECM elements. The preservation of cellular integrity, heterogeneity and ECM niche within cerebroids makes this model uniquely suited for investigating normal developmental processes and cell–cell interactions, as well as disruptions caused by environmental insults.

Utilizing the cerebroid, we demonstrated that IL-17A induces cortical abnormalities associated with MIA, including premature cortical folding, increased thickness and accelerated neuronal maturation, driven by enhanced neurogenesis in NSCs. Modeling MIA in cerebroids has revealed clinically relevant cellular and molecular phenotypes associated with NDDs, such as microglia-driven immune responses, structural cortical changes, an increased number of excitatory neurons and alterations in the ECM^22–25^. Furthermore, this model offers a unique opportunity to dissect the molecular and mechanistic basis of these abnormalities in humans during early development, a stage that is typically inaccessible. Our transcriptomic and proteomic analyses revealed that IL-17A remodels the ECM, with contributions from various cortical cell types.

Despite the essential role of the ECM in key developmental processes^20,37^, its involvement in various NDDs has been relatively underexplored. Our findings, which demonstrate ECM remodeling in response to MIA in ex vivo cultured human fetal brain tissues, alongside the altered expression of ECM-related genes observed in post-mortem brains of individuals with ASD, highlight the critical need for a comprehensive investigation into the molecular mechanisms underlying ECM alterations and their developmental implications in the human brain.

A central advance of this study is the demonstration that IL-17A directly activates NF-κB signaling in human NSCs. We show that IL-17 receptors are expressed in NSCs, are upregulated upon IL-17A exposure and engage downstream signaling cascades involving ACT1. Transcriptomic profiling revealed upregulation of *TRAF3IP2* and downregulation of the NF-κB inhibitors, *TNFAIP3* and *NFKBIB*, indicating sustained inflammatory activation. Proteomics further identified NF-κB-associated activators such as TRAF6 and TRAF2, while transcription factor enrichment confirmed a robust NF-κB activation signature. These findings were functionally validated in human iPSC-derived NPCs, where IL-17A induced prolonged phosphorylation and nuclear translocation of p65, in contrast to the transient response triggered by TNF. Crucially, this sustained NF-κB activation, along with increased phospho-p65 expression, was rescued by the NF-κB inhibitor PTL. Additionally, PTL treatment successfully reversed IL-17A-induced cortical folding and neurogenesis abnormalities as well as aberrant upregulation of BCAN expression in cerebroids, implicating NF-κB signaling involvement. Together, these multimodal data establish NF-κB signaling as a mechanistic driver of IL-17A-induced abnormalities in human cortical development.

Our study extends prior findings from rodent models and human NPC studies by demonstrating that IL-17A directly engages its receptors on human fetal cortex-derived NSCs, induces sustained NF-κB signaling and remodels ECM composition. Whereas earlier work in human NPCs identified ERK/mTOR pathways as IL-17A targets without evidence of NF-κB activation^51^, our multimodal approach reveals a broader signaling repertoire with functional consequences for cortical architecture. These results advance understanding of how MIA perturbs human corticogenesis and provide a platform for dissecting molecular pathways that may underlie NDD risk.

Epidemiological, clinical and animal studies have shown that males are more vulnerable to NDDs associated with MIA, both in terms of prevalence and symptom manifestation^52^. Sex differences in response to MIA are likely influenced by a complex interplay of hormonal, placental and microglia-mediated inflammation^53^, although the precise mechanisms remain unclear. Due to limited tissue availability, sex differences were not explored in this study and further research is needed to address this gap.

In this study we cultured cerebroids for 15 d at most. A recent study demonstrated that fetal brain organoids can be maintained in long-term culture through continuous tissue fragmentation^54^. However, maintaining proper cytoarchitectural polarity remains a major challenge, emphasizing the need for future efforts to optimize long-term cerebroid culture protocols to preserve normal developmental trajectory, cytoarchitecture and cellular integrity. Achieving this will enable more comprehensive, long-term investigations into the impact of environmental factors on cortical development. Nonetheless, cerebroids provide a robust platform for investigating the acute effects of environmental insults.

In conclusion, we have established cerebroids as a robust, physiologically relevant model for interrogating complex neurodevelopmental processes. We have also demonstrated their application as a platform to study IL-17A-mediated disruptions in cortical development during MIA. In addition to incorporating neuronal and glial elements, cerebroids also integrate immune and vascular components, along with the ECM. This physiologically relevant complexity offers a unique platform to explore cell–cell and cell–ECM interactions across diverse cell populations, providing valuable insights into healthy brain development and NDDs.

## Methods

### Ethics statement

All research involving human fetal tissue was conducted in accordance with relevant ethical regulations and the principles of the Declaration of Helsinki. Human fetal tissue was obtained through the Scottish Advanced Fetal Research (SAFeR) study (NCT04613583), which was approved by the Scottish National Health Service Research Ethics Committee (REC 15/NS/0123). Written, informed consent was obtained from all participants before tissue donation. Human iPSC and NPC lines were obtained from commercial sources and used in accordance with relevant ethical regulations. The stem cell aspects of this study are consistent with the principles outlined in the 2021 ISSCR Guidelines for Stem Cell Research and Clinical Translation.

### Human fetal tissue collection

Fetal tissues from elective termination of normally progressing pregnancies were collected as part of the SAFeR study (NCT04613583)^55^. The collection process is approved by Scottish National Health Service Research Ethics Committee (REC 15/NS/0123) and follows the Declaration of Helsinki guidelines. Women seeking elective medical termination of pregnancy were recruited with written, informed consent by NHS Grampian research nurses, who operate independently from the research team. Participants received a Patient Information Sheet at least 24 h before consent was obtained and provided written, informed consent before enrollment in the study. The normal pregnancies were determined by ultrasound, from women over 16 years of age who spoke English and covering gestational ages from 7 to 20 weeks. Grossly abnormal fetuses were excluded, and women experiencing substantial distress were not approached. Termination was carried out using RU-486 (Mifepristone) treatment (200 mg) followed by prostaglandin-induced delivery, as reported previously^56^. Gestational age was confirmed by ultrasound and foot length measurement, and various maternal and fetal data were recorded. For sample collection, fetuses were immediately transported to the laboratory (Extended Data Fig. 1a), typically intact, and were weighed, measured for crown–rump length and sexed by morphology and PCR detection of sex chromosome-specific genes (*ZFX* and *SRY*) or the absence of the Y chromosome. Brain tissues from 16 donors of 11 to 14 weeks of gestation collected between 11 May 2022 and 22 April 2024 were included in this study (Supplementary Table 1). Tissue samples used in this study were consumed during experimental procedures and are no longer available for distribution.

### Brain dissection and tissue culture

Whole brain tissue was transferred to cold Hanks’ balanced salt solution (HBSS) (Gibco, cat. no. 14175095) on ice for dissection. The pia matter was carefully removed. Full-thickness, approximately 10-mm^2^-sized pieces of right DLPFC tissue were dissected and plated in six-well tissue culture plates (16–24 cerebroids per well). Dissection time was kept to a minimum (~30 min) and cerebroids were immediately transferred to the fresh culture medium (4 ml per well) containing a 1:1 mixture (v/v) of Dulbecco’s Modified Eagle Medium/Nutrient Mixture F-12 (DMEM/F-12, Gibco, cat. no. 11330-032) and Neurobasal Medium (Gibco, cat. no. 21103-049), supplemented with 1 × GlutaMAX (Gibco, cat. no. 35050-038), 1 × MEM nonessential amino acids Solution (Gibco, cat. no. 11140-050), 1 × serum-free B27 (Gibco, cat. no. 17504-044), 1 × N-2 (Gibco, cat. no. 17502-048), 1 × 2-mercaptoethanol (Gibco, cat. no. 21985-023), 2.5 μg ml^−1^ human recombinant insulin (Gibco, 12585-014) and 1 × antibiotic-antimycotic (Gibco, cat. no. 15240-096). Cerebroids were cultured in a free-floating manner on a CO_2_-resistent orbital shaker (Fisher Scientific, cat. no. 15351105) at a rotational rate of 120 per min in a humidified incubator (37 °C and 5% CO_2_). Culture medium was changed on alternate days.

### Cell culture

The human iPSC line KOLF2.1J purchased from The Jackson Laboratory was used to generate cortical organoids and iPSC-derived microglia-like cells. Human iPSCs were cultured in mTeSR Plus Medium containing mTeSR Plus Basal Medium (StemCell, cat. no. 100-0274), mTeSR Plus 5X Supplement (StemCell, cat. no. 100-0275) and 1 × penicillin–streptomycin (Gibco, cat. no. 15140-122).

Human iPSC line SCTi003-A-derived NPCs (StemCell, cat. no. 200-0620) were cultured in STEMdiff neural progenitor medium (StemCell, cat. no. 05833) supplemented with 1 × penicillin–streptomycin according to the supplier’s instructions.

NPCs were differentiated into forebrain neurons using STEMdiff Forebrain Neuron Differentiation Medium (StemCell, cat. no. 08600) in Laminin/Poly-L-Ornithine (Sigma-Aldrich, cat. no. LPLO001)-coated 12-well plates followed by maturation in STEMdiff Forebrain Neuron Maturation Medium (StemCell, cat. no. 08605).

Human iPSC-derived microglia-like cells were generated from Kolf2.1J cells using a previously published protocol^57^.

### iPSC-derived cortical organoid culture

Human KOLF2.1J iPSC-derived cortical organoids were generated using previously published protocols^58,59^. iPSCs were seeded into ultra-low-attachment 96-well plates (Corning, cat. no. 7007) at 5 × 10^4^ cells per well in mTeSR-based medium supplemented with ROCK inhibitor (1:2,000), followed by centrifugation (300*g*, 5 min, 4 °C) and incubation at 37 °C and 5% CO_2_ for 48 h to form embryoid bodies. At day 2, embryoid bodies were transferred (~24 per well) to ultra-low-attachment six-well plates (Corning, cat. no. 3471) and cultured on an orbital shaker (120 rpm) in neural induction medium consisting of DMEM/F12 (Gibco, cat. no. 11330-032), 20% KnockOut Serum Replacement (Gibco, cat. no. 10828-028), GlutaMAX (Gibco, cat. no. 35050-038), nonessential amino acids (Gibco, cat. no. 11140-050), 2-mercaptoethanol (Gibco, cat. no. 21985-023), penicillin–streptomycin, heparin (StemCell, cat. no. 7980) and dual SMAD inhibitors SB431542 (StemCell, cat. no. 72234; 5 µM) and LDN-193189 (StemCell, cat. no. 72147; 1 µM), with ROCK inhibitor included for an additional 48 h. Medium was refreshed daily for days 4–5, and from day 6 cultures were transitioned to a patterning medium containing DMEM/F12, N2 supplement (Gibco, cat. no. 17502-048), GlutaMAX, nonessential amino acids, 2-mercaptoethanol, penicillin/streptomycin and CHIR99021 (StemCell Technologies, cat. no. 72054; 3 µM). At day 7, morphologically healthy embryoid bodies were embedded in Matrigel in a 2:3 mixture with medium and plated as droplets, allowed to polymerize at 37 °C and cultured for 48 h in patterning medium without agitation, with partial medium changes every other day for days 9–13. At day 14, organoids were mechanically released from Matrigel, washed with phosphate buffered saline (PBS) and transferred to maturation medium consisting of a 1:1 mixture of DMEM/F12 and Neurobasal medium (Gibco, cat. no. 21103-049) supplemented with N2, B27 (Gibco, cat. no. 17504-044), GlutaMAX, nonessential amino acids, penicillin–streptomycin, 2-mercaptoethanol, insulin (Gibco, cat. no. 12585-014) and CHIR99021 (1.5 µM), and maintained on an orbital shaker (120 rpm) with half-medium changes every other day.

### IL-17A treatment

MIA was modeled by treating cerebroids and organoids with 100 ng ml^−1^ human recombinant IL-17A (PeproTech, cat. no. 200-17) every other day for a week (Figs. 2a and 3k). The dose was chosen based on the previous in vivo^60–64^ and two-dimensional (2D)^61,65,66^ and 3D^62,67,68^ in vitro studies. The dose of IL-17A used in earlier studies has been variable, ranging from 10 ng ml^−1^ to 500 ng ml^−1^ in 2D and 25 ng ml^−1^ to 500 ng ml^−1^ culture medium in 3D in vitro cultures. We added 100 ng ml^−1^ IL-17A to the cerebroid and organoid culture every 48 h. For both vehicle- and IL-17A-treated groups, two-thirds of the medium was replaced with fresh medium containing vehicle or IL-17A. 2D cultured NPCs were treated with 12.5 ng ml^−1^ IL-17A either daily for 5 d (Fig. 3n) or 2 d (Fig. 5h–k), or as single treatment for 30 min and 24 h (Fig. 5i–l). NPC-derived forebrain neurons were treated with 12.5 ng ml^−1^ IL-17A every 2 d for 1 week (Extended Data Fig. 4a).

### TNF treatment

NPCs from 2D cultures were treated with a single dose of 25 ng ml^−1^ TNF (PeproTech, cat. no. 300-01A-50UG) for 30 min and 24 h.

### PTL treatment

Cerebroids were treated with 25 µM (-)-PTL (Selleckchem, cat. no. S2341) in cerebroid culture medium on alternative days for a week with and without IL-17A treatment (Fig. 5b). We used 0.2% DMSO as vehicle control. 2D NPCs were treated with 10 µM PTL in combination with 12.5 ng ml^−1^ IL-17A (Fig. 5o,p).

### Tissue preparation and immunofluorescent staining

Cerebroids were fixed in 4% paraformaldehyde (PFA) in PBS for 60 min at room temperature and cryoprotected with 30% sucrose overnight at 4 °C. Fetal brain tissue was fixed in 4% PFA in PBS overnight and cryoprotected sequentially with 15% and 30% sucrose at 4 °C. Cortical organoids were fixed in 4% PFA in PBS for 30 min at room temperature. Cerebroids and fetal brain tissue were cryo-sectioned at −20 °C in a cryostat (Leica Biosystems, cat. no. CM1850) perpendicular to the ventricular surface at 20-μm thickness, and stored at −20 °C. Frozen slides were gradually equilibrated to room temperature before immunostaining. For each set of staining, a minimum of 3–4 sections 160–200 μm apart were included per cerebroid. After washing and permeabilization with TBS+ (0.05 M Tris-buffered saline, 0.1% Triton-X, pH 9.0) and antigen retrieval with 1 × Target Retrieval Solution (Dako Agilent, cat. no. S169984-2) at 88–90 °C for 15 min in a plastic Coplin staining jar (Epredia, cat. no. 194), sections were blocked with the blocking solution TBS++ (containing 10% donkey serum in TBS with 0.1% Triton-X) for 1 h at room temperature. Tissue sections were incubated with primary antibodies (Supplementary Table 14) diluted in TBS with 0.1% Triton-X and 3% donkey serum overnight at 4 °C in an immunostaining moisture chamber (Agar Scientific, cat. no. AGL4555). A longer incubation time (48–72 h) was used for primary staining with anti-HOPX antibodies. After washing, tissues were incubated with species-specific secondary antibodies (Supplementary Table 14) and DAPI (1:5,000) diluted in TBS+ with 3% donkey serum for 2 h at room temperature.

NPCs cultured on chamber slides were fixed with 4% PFA in PBS at room temperature for 15 min.

For WGA staining of fixed tissue, cerebroid sections were washed with PBS without permeabilization agents, and incubated with Alexa Fluor 555-conjugated WGA (Invitrogen, cat. no. 11590816) in PBS at 10 µg ml^−1^ for 30–60 min at room temperature.

### EdU pulse–chase experiments

For EdU pulse–chase experiments, 10 μM EdU (Invitrogen, cat. no. A10044) was added to culture medium for 2 h (Figs. 1g and 3a).

EdU detection was done after incubation with the secondary antibodies using the Click-iT EdU cell proliferation kit for imaging and Alexa Fluor 647 dye (Invitrogen, cat. no. C10340) according to the manufacturer’s instructions.

### Genetic labeling of radial glia cells using adenovirus

The recombinant human Adenovirus Type 5 (dE1/E3)-expressing enhanced green fluorescent protein (Vector Biolabs, cat. no. 1060) or red fluorescent protein (Vector Biolabs, cat. no. 1660) under CMV promoter was used to selectively label apical radial glia cells in the cerebroids. We added ~15–20 µl of DMEM/F12 containing 1 × 10^7^ plaque forming units per µl of adenovirus directly on the ventricular surface of the cerebroids after removing the culture medium. Cerebroids were incubated for 20–30 min at 37 °C and 5% CO_2_ without agitation. Cerebroids were then washed three times with DPBS and cultured in the fresh medium.

### Calcium imaging

Neurons were incubated with 1:1 v/v 10 µM Fluor-4-AM (Fisher Scientific, cat. no. 11504786) and Pluronic acid (Fisher Scientific, cat. no. P3000MP) in the culture medium for 30 min in the incubator. Cells were washed three times with HBSS without calcium and magnesium and then incubated with HBSS containing calcium and magnesium for 20 min at room temperature. Cells were imaged on the EVOS M5000 imaging system (Thermo Fisher, cat. no. AMF5000SV) with the EVOS onstage incubator (Thermo Fisher, cat. no. AMC2000) with the following settings: 37 °C, 5% CO_2_ and 80% humidity.

Images were analyzed using ImageJ. Movement artifacts were removed using Linear Stack Alignment with SIFT plugin and photo bleach correction performed using the exponential bleach correction function. Circular regions of interest (ROIs) were drawn for individual neurons and fluorescence intensities were measured across the stack. ∆*F*/*F*_min_ values were calculated and plotted in GraphPad Prism. Heatmaps were drawn in R using ComplexHeatmap package v2.20.0 (https://bioconductor.org/packages/release/bioc/html/ComplexHeatmap.html).

### Immunofluorescence image acquisition and image analysis

Images were acquired on a laser scanning confocal microscope (Carl Zeiss, cat. no. LSM880) using ×20, ×40 (oil immersion) and ×63 (oil immersion) objectives. Uniform laser settings were used across experimental groups where intensity-based analysis was planned. For uniformity, images were obtained from the central third of the cerebroid sections. At least two to three sections were imaged for each cerebroid.

Images were analyzed using Fiji ImageJ software. To achieve consistent and reproducible results, we applied a uniform image acquisition, pre-processing and analysis pipeline to count cells across the experimental groups for a given set of staining. Rectangular ROIs were drawn with the base of the rectangle parallel to the ventricular surface and length covering the whole thickness of the cerebroid section to include all layers (Extended Data Fig. 3f,g). The width of these ROIs was kept constant for comparison across experimental groups and replicates. For quantification of nuclei positive for nuclear markers (for example, SOX2 and CTIP2), images were segmented through ImageJ interactive watershed plugin version 1.2.1 (https://imagej.net/plugins/interactive-watershed) and binary masks were generated. Uniform settings (seed dynamics, threshold intensity and peak flooding) were used for segmenting nuclei across experimental groups for each set of staining. Numbers of binarized nuclei were quantified using the analyze particle function in ImageJ. For all quantitative analysis, images were blinded before quantification.

No preprocessing was performed for intensity-based quantification analysis (Fig. 2m,n). For nuclear fluorescence intensity measurement of different markers, nuclei were segmented from DAPI images using interactive watershed plugin version 1.2.1 (Extended Data Fig. 3h), and individual masks were generated for these nuclei and applied to the 8-bit images of various markers (for example, CTIP2, SATB2 and NeuN). We measured the mean fluorescence intensity (MFI) for these individual nuclei. Nuclei less than 8 or greater than 200 µm in size were filtered out. Fluorescence intensities were corrected for background noise by subtracting average background intensities.

Nuclear and cytoplasmic MFI were quantified by applying compartment-specific masks. Nuclear masks were generated using SOX2 staining, and cytoplasmic masks were generated using Nestin staining. ACT1 fluorescence intensity was measured within cytoplasmic masks, while phosphorylated p65 fluorescence intensity was measured within both nuclear and cytoplasmic masks.

### Measurement of cortical folding

Cortical folding was quantified by measuring GI (Fig. 2f) on low-magnification (×4 or ×10) DAPI images of cerebroids.

GI was calculated as follows:

GI = length of fiducial (inner) surface/length of (outer) convex hull

### Measurement of layer thickness

Total thickness of a cerebroid was measured as an average of three vertical lines drawn perpendicular to the ventricular surface through the central part of the DAPI-stained images of a cerebroid. Three layers of cerebroids (layers 1–3) were defined based on the cell densities (layer 1 and layer 3 being cell-dense, layer 2 being cell-sparse) and staining of specific markers (SOX2- and TBR2-enriched layer 1, CTIP2-enriched layer 3) (Fig. 2e and Extended Data Fig. 3f,g).

### Electron microscopy

Tissue was fixed in 2% glutaraldehyde (TAAB, cat. no. G010) and 4% formaldehyde (Fisher Scientific, cat. no. 28909) in 0.1 M cacodylate (TAAB, cat. no. S007) buffer for 48 h at 4 °C and stored in 0.1 M cacodylate buffer at 4 °C until further processing. Samples were postfixed with 1% osmium tetroxide (TAAB, cat. no. O021) and 1.5% potassium ferrocyanide (Sigma, cat. no. P-3289) in 0.1 M cacodylate buffer, washed and sequentially dehydrated with 35%, 50%, 70% and 95% ethanol followed by acetone. This was followed by Spurr’s infiltration and embedding in Spurr’s resin for 48 h at 60 °C. Sections 90-nm thick were prepared using a diamond knife (Diatome) onto formvar/carbon-coated copper grids (EMResolutions) using a Leica UC6 ultramicrotome and contrast stained with Uranyless (Labtech) and lead citrate (Leica) using a Leica AC20. Samples were viewed by TEM on the JEM-1400Plus (JEOL) and images were captured using an UltraVUE camera (AMT). SEM images were acquired on a ZEISS EVO MA10.

### WGA staining for live tissue

For live staining with Alexa Fluor 555-conjugated WGA (Invitrogen, cat. no. 11590816), cerebroids were transferred to the wells of a 12-well plate containing WGA diluted in HBSS at 10 µg ml^−1^ for 30 min at room temperature. Cerebroids were washed three times with HBSS, imaged and transferred back to the fresh culture medium.

### Assessment of the basal inflammation level of the donor fetal tissue

RNA was extracted from snap-frozen and pulverized placental tissue and reverse-transcribed into complementary DNA using the GoScript Reverse Transcriptase Kit (Promega, 2790). Primers for *C3*, *TNF*, *IL6*, *IL1B* and *NOS2* were designed using Integrated DNA Technologies PCR designing web tool (https://www.idtdna.com/page) (Supplementary Table 15). Transcript expression was quantified by reverse transcription quantitative PCR (RT–qPCR) using the LightCycler 480 System and LightCycler 480 SYBR Green I Master (Roche). Samples were run in triplicate against a six-point standard curve for each transcript (Absolute Quantification analysis) using the following parameters: 95 °C for 5 min, 45 cycles of 95 °C for 15 s, 63 °C for 15 s and 72 °C for 10 s, followed by a melting curve.

### Tissue dissociation and single-cell isolation

Cerebroids were dissociated into single cells through enzymatic digestion and mechanical dissociation using a previously published protocol^69^ with the following modifications: the myelin removal step with the Percoll method was not required and the duration of enzymatic digestion and mechanical dissociation was kept at 15 min.

### Immunomagnetic cell sorting

Microglia were isolated through immunomagnetic positive selection of CD11b^+^ cells by using EasySep Human CD11b Positive Selection and Depletion Kit (StemCell, cat. no. 100-0742) and The Big Easy EasySep Magnet (StemCell, cat. no. 18001) according to the manufacturer’s instructions. CD24^+^THY1^−^CD11b^−^ and CD24^−^THY1^−^CD11b^−^ cells representing NLCCs and NSCs, respectively^29^, were isolated from a CD11b-depleted single-cell population. CD11b^−^ cells were subjected sequentially to immunomagnetic sorting with biotinylated anti-THY-1 (CD90) antibodies (StemCell, cat. no. 60045BT) and anti-CD24 antibodies (Miltenyi Biotec, cat. no. 130-098-902) using the EasySep Release Human Biotin Positive Selection Kit (StemCell, cat. no. 17653) and EasySep Magnet according to the manufacturer’s guidelines (Extended Data Fig. 5a).

### RNA extraction and RNA-seq

Total RNA was extracted from whole cerebroids and isolated cells using the RNeasy mini kit (Qiagen, cat. no. 74104) according to the manufacturer’s instructions in an RNase-free environment. Residual genomic DNA was removed by on-column digestion with RNase-free DNase I (Qiagen, cat. no. 79254). RNA quantity was measured on a Qubit Fluorimeter and RNA integrity number on an Agilent TapeStation. cDNA libraries were prepared with 50 ng of input at 14 cycles of amplification using Illumina Stranded mRNA Prep, Ligation kit (cat. no. 20040532) and sequenced on a NextSeq 500 machine.

FASTQ files of single-end reads were processed using FASTP^70^. Processed reads were mapped to human reference genome assembly GRCh38 and transcript-level quantification was obtained using Salmon v1.10.2^71^. Transcript-level quantification data were imported in R, and gene-level quantification was obtained using tximeta^72^. Differential gene expression was estimated using DESeq2 package^73^. Volcano plots were generated using ggplot2 (version 3.5.1) and ggrepel (version 0.9.6) packages.

Heatmaps were generated using the pheatmap package. Read counts were normalized with DESeq2 and variance-stabilizing-transformed. *Z*-scores were calculated for each gene across samples, and mean *Z*-scores from three donors per group were plotted.

### Protein fractionation and proteomic analysis

For proteomic analysis using liquid chromatography–tandem mass spectrometry (LC–MS/MS), vehicle- and IL-17A-treated day 8 cerebroids were collected from three donors and snap-frozen in liquid nitrogen. Protein extraction, fractionation and LC–MS/MS (Extended Data Fig. 8a) were performed using a previously published three-step decellularization and ECM extraction protocol that yields cellular (CHAPS), sECM and iECM fractions^74^.

Protein-level abundance quantification data were imported in R. For each fraction, proteins with nonzero abundance in at least two samples were included in the analysis. Protein abundance values were log-transformed and normalized with the median centered method of data normalization. Missing values were imputed by random draws from a Gaussian distribution centered to a minimal value using the impute.MinProb() command in the imputeLCMD package v2.1. The SummarizedExperiment package was used to generate a summarized experiment object; the limma^75^ package (version 3.19) was used to identify differentially abundant proteins by applying a multifactorial design assay including the treatment group and donor.

### Meta-analysis of RNA-seq data

For the meta-data of post-mortem brains of individuals with ASD, we searched PubMed and Web of Science for studies published in English, from database inception to 30 May 2023, that investigated the transcriptome of human ASD brains. We used the search terms (‘ASD’ OR ‘autism’) AND (‘brain’ OR ‘cortex’) AND (‘transcriptome’ OR ‘RNAseq’ OR ‘microarray’ OR ‘gene expression’). This led to identification of three microarray^76–78^, six bulk^31–33,79–81^ and three single-nuclear RNA-seq^33,82,83^ studies on the post-mortem brain tissue of ASD cases and controls. Data from one study^80^, after applying the filters, gave a list of only six upregulated genes (two mitochondrial genes and four small nucleolar RNA genes) and were excluded from further analysis. Studies that included bulk RNA-seq data from the frontal cortex^31–33^ and meta-analysis of microarray data^32^ were selected for further analysis. To identify functionally enriched terms and pathways in an unbiased manner, we applied a protein sub-network-based functional enrichment analysis using STRING v12.0^84^. Functional enrichment analysis was performed for each individual study, with genes ranked according to log_2_ fold change in ASD versus control brains. A list of all enriched pathways for four studies is provided in Supplementary Table 10. Terms enriched in at least two independent studies, with 50 or more genes and with a false discovery rate (FDR)-adjusted *P* < 0.05, were considered significant and plotted (Extended Data Fig. 7).

For analyses of BCAN and IL-17 receptor expression, we used previously published single-cell RNA-seq metadata^45^ from 37 donors spanning gestational weeks 8 to 26 across six independent studies^85–90^. Gene expression for each cell type within each donor was aggregated into a pseudobulk profile, with one cell type per donor treated as a single observation.

### Functional enrichment analysis

For transcriptomic and proteomic data, we applied a protein sub-network-based functional enrichment analysis using STRING v12.0 as described above. *K*-means clustering was performed on the protein–protein interaction network of differentially abundant proteins using STRING.

Overrepresentation analysis was performed using Enrichr^91^ (https://maayanlab.cloud/Enrichr/). Enrichr was used to identify a list of enriched transcription factors having regulatory interactions with DEGs in the human TRRUST v2 (Transcriptional Regulatory Relationships Unraveled by Sentence-based Text mining) reference database^92^.

The list of genes implicated in neurological disorders is adopted from a recent study^93^.

### Statistical analysis and data visualization

No statistical methods were used to pre-determine sample sizes. Sample sizes were determined by the availability of human fetal tissue and practical considerations associated with the experimental procedures. GraphPad Prism (version 8.4.3) and the R (version 4.3.1) statistical computing environment (https://www.r-project.org/) were used to analyze data and generate graphs. Samples were randomly assigned to experimental groups. Data normality was formally assessed using the Shapiro–Wilk test and Q–Q plots, and homogeneity of variance was assessed before statistical analysis where appropriate. Data collection and analysis were performed blind to the experimental conditions. Two-way analysis of variance (ANOVA) test was applied to compare across experimental groups (vehicle- versus IL-17A-treated) and donors. For inter-group comparison across multiple groups, Tukey’s multiple comparisons test was performed. Donor-level quantification is represented as mean ± s.e.m. while individual data points represent technical replicates. No data point was excluded from all the analyses. On average, 2–3 sections per cerebroid and 2–3 cerebroids per donor per group were analyzed for each quantification. The numbers of donors for each quantification are provided in the figure legends.

Plots were made in GraphPad Prism or R and edited in Inkscape v 1.3.2.

### Reporting summary

Further information on research design is available in the Nature Portfolio Reporting Summary linked to this article.

## Online content

Any methods, additional references, Nature Portfolio reporting summaries, source data, extended data, supplementary information, acknowledgements, peer review information; details of author contributions and competing interests; and statements of data and code availability are available at https://doi.org/10.1038/s41593-026-02400-2.

## Supplementary information

Reporting Summary

Supplementary Information Supplementary Tables 1–15.

## Source data

Source Data Statistical source data for Figs. 1–6 and Extended Data Figs. 3–5, 7 and 9.

## Data Availability

All data are available in the main text or the supplementary information. RNA-seq data have been deposited at the NCBI GEO (GSE280371). The mass spectrometry proteomics data have been deposited to the ProteomeXchange Consortium via the PRIDE partner repository with the dataset identifier PXD070178. Source data are provided with this paper.

## Code availability

All code has been deposited and is available via GitHub at https://github.com/KANGBERGLAB/MIA_Assir/.
