# Supplementary material for: Modeling maternal immune activation in 3D ex vivo human fetal brain cerebroids reveals IL-17A-driven disruption of cortical development
Source: Nat Neurosci. 2026 Aug 11;29(10):2456–68. doi: 10.1038/s41593-026-02400-2 (PMC13633116; doi:10.1038/s41593-026-02400-2)
Supplement: Supplementary file 1 [file 41593_2026_2400_MOESM1_ESM.pdf]

Corresponding author(s): Eunchai Kang

Last updated by author(s): May 28, 2026

Reporting Summary

Nature Portfolio wishes to improve the reproducibility of the work that we publish. This form provides structure for consistency and transparency in reporting. For further information on Nature Portfolio policies, see our Editorial Policies and the Editorial Policy Checklist.

Statistics

For all statistical analyses, confirm that the following items are present in the figure legend, table legend, main text, or Methods section.

n/a	Confirmed	
☐	☒	The exact sample size (*n*) for each experimental group/condition, given as a discrete number and unit of measurement
☐	☒	A statement on whether measurements were taken from distinct samples or whether the same sample was measured repeatedly
☐	☒	The statistical test(s) used AND whether they are one- or two-sided<br>*Only common tests should be described solely by name; describe more complex techniques in the Methods section.*
☐	☒	A description of all covariates tested
☐	☒	A description of any assumptions or corrections, such as tests of normality and adjustment for multiple comparisons
☐	☒	A full description of the statistical parameters including central tendency (e.g. means) or other basic estimates (e.g. regression coefficient) AND variation (e.g. standard deviation) or associated estimates of uncertainty (e.g. confidence intervals)
☐	☒	For null hypothesis testing, the test statistic (e.g. *F*, *t*, *r*) with confidence intervals, effect sizes, degrees of freedom and *P* value noted<br>*Give P values as exact values whenever suitable.*
☒	☐	For Bayesian analysis, information on the choice of priors and Markov chain Monte Carlo settings
☒	☐	For hierarchical and complex designs, identification of the appropriate level for tests and full reporting of outcomes
☒	☐	Estimates of effect sizes (e.g. Cohen's *d*, Pearson's *r*), indicating how they were calculated

Our web collection on statistics for biologists contains articles on many of the points above.

Software and code

Policy information about availability of computer code

Data collection	Immunofluorescence image data was collected using Zen in Carl Zeiss LSM880 and software installed in EVOS M5000.<br>Transmission Electron Microscopy images were obtained JEM 1400 plus (JEOL UK) and an AMT UltraVUE camera (AMT, USA).<br>Scanning electron microscopy images were acquired on ZEISS EVO MA10 SEM.<br>Calcium imaging was obtained using software installed in EVOS M5000<br>RNA quantity was measured on a Qubit Fluorimeter and RNA integrity number (RIN) on an Agilent TapeStation.<br>RNA sequencing data was collected using a NextSeq 500 platform.<br>Other data were collected in MS office.<br>Meta data of autism spectrum disorder transcriptomic data was collecged through PubMed and Web of Science.
Data analysis	Immunofluorescence images were analyzed using Fiji ImageJ software. images were segmented through ImageJ interactive watershed plugin version 1.2.1<br>Calcium imaging was analyzed using ImageJ and movement artifacts were removed using Linear Stack Alignment with SIFT plugin.<br>For RNA-sequencing data analysis, FASTQ files of single-end reads were processed using FASTP.<br>Processed reads were mapped to human reference genome assembly GRCh38 and transcript level quantification was obtained using Salmon v1.10.2.<br>Transcript-level quantification data was imported in R, and gene-level quantification was obtained using tximeta,.<br>Differential gene expression was estimated using DESeq2 package.<br>Volcano plots were generated using ggplot2 (version 3.5.1) and ggrepel (version 0.9.6) packages.<br>Proteomic data analysis was done in R using imputeLCMD package v2.1, SummarizedExperiment package, and limma package (version 3.19)<br>Functional enrichment analysis was done using STRING v12.0.<br>Enrichr was used to identify list of enriched transcription factors having regulatory interactions with DEGs in human- TRRUST v2 reference

database.
All statistical analysis was done in GraphPad Prism (version 8.4.3) and R (version 4.3.1)
Plots were made in GraphPad Prism or R and edited in Inkscape v 1.3.2.

For manuscripts utilizing custom algorithms or software that are central to the research but not yet described in published literature, software must be made available to editors and reviewers. We strongly encourage code deposition in a community repository (e.g. GitHub). See the Nature Portfolio guidelines for submitting code & software for further information.

Data

Policy information about availability of data

All manuscripts must include a data availability statement. This statement should provide the following information, where applicable:

- Accession codes, unique identifiers, or web links for publicly available datasets
- A description of any restrictions on data availability
- For clinical datasets or third party data, please ensure that the statement adheres to our policy

All data are available in the main text or the supplementary information and supporting data file.
All original code has been deposited at GitHub: https://github.com/KANGBERGLAB/MIA_Assir/.
RNA-seq data has been deposited at the NCBI GEO: GSE280371.
The mass spectrometry proteomics data have been deposited to the ProteomeXchange Consortium via the PRIDE partner repository with the dataset identifier PXD070178.

Research involving human participants, their data, or biological material

Policy information about studies with human participants or human data. See also policy information about sex, gender (identity/presentation), and sexual orientation and race, ethnicity and racism.

Reporting on sex and gender	The terms 'male' and 'female' used in this manuscript refer to sex or biological attributes. The sex of all fetal brain donors is listed in Supplementary Table 1.
Reporting on race, ethnicity, or other socially relevant groupings	n/a
Population characteristics	n/a
Recruitment	Women seeking elective medical termination of pregnancy were recruited with written informed consent by NHS Grampian research nurses, who operate independently of the research team. Normal pregnancies were confirmed by ultrasound, and participants included women over 16 years of age who speak English, with gestational ages ranging from 7 to 20 weeks. Grossly abnormal fetuses were excluded, and women experiencing significant distress were not approached.
Ethics oversight	Fetal tissues from elective terminations of normally progressing pregnancies were collected as part of the Scottish Advanced Fetal Research (SAFeR) study (NCT04613583)[1]. The collection process was approved by one of the 12 Scottish National Health Service Research Ethics Committees (REC 15/NS/0123) and complies with the Declaration of Helsinki guidelines

Note that full information on the approval of the study protocol must also be provided in the manuscript.

Field-specific reporting

Please select the one below that is the best fit for your research. If you are not sure, read the appropriate sections before making your selection.

☒ Life sciences ☐ Behavioural & social sciences ☐ Ecological, evolutionary & environmental sciences

For a reference copy of the document with all sections, see nature.com/documents/nr-reporting-summary-flat.pdf

Life sciences study design

All studies must disclose on these points even when the disclosure is negative.

Sample size	We performed a two-way ANOVA since we had two variables: conditions (vehicle vs. IL-17A) and donors. We calculated the effect size (partial eta squared) for three different measurements: thickness, the number of CTIP2+ nuclei (Fig. 2g, i), and the cortical folding index (Fig. 2f). The effect size was approximately 0.9 for all three measurements. Using the G*Power program, we generated a plot of power as a function of total sample size with a Type I error rate of 5%. This analysis indicated that at least 17–20 samples are required to achieve 80% power. Consequently, we tried to analyze at least 2-3 sections from 2-6 cerebroids from 2–8 donors .
Data exclusions	No data was exclude from the analyses.
Replication	All experiments were repeated using two to eight different biological replicates/independent samples (from different tissue donors) and multiple technical replicates. The number of cerebroids and donors is specified in the respective figure legends.

Randomization	We randomly distributed the cells/cerebroids onto cell culture dishes or multi-well plate positions and randomly assigned them to experimental groups.
Blinding	All quantifications were performed in a blinded manner.

Reporting for specific materials, systems and methods

We require information from authors about some types of materials, experimental systems and methods used in many studies. Here, indicate whether each material, system or method listed is relevant to your study. If you are not sure if a list item applies to your research, read the appropriate section before selecting a response.

Materials & experimental systems

n/a	Involved in the study	
☐	☒	Antibodies
☐	☒	Eukaryotic cell lines
☒	☐	Palaeontology and archaeology
☒	☐	Animals and other organisms
☒	☐	Clinical data
☒	☐	Dual use research of concern
☒	☐	Plants

Methods

n/a	Involved in the study	
☒	☐	ChIP-seq
☒	☐	Flow cytometry
☒	☐	MRI-based neuroimaging

Antibodies

Antibodies used

BM28/MCM2 Mouse IgG BD Biosciences - #610701
CD68 Mouse IgG Sigma - #AMAB90874
Cleaved Caspase-3 Rabbit IgG Cell Signalling Technology - #9661
Ctip2 Rat IgG2a Abcam - #Ab18465
EOMES/Tbr2 Sheep IgG R&D Systems #AF6166
GFAP Mouse IgG Millipore - #MAB360-25UL
GFAP Chicken IgY Merck - AB5541
GFP Goat IgG Rockland - #600-101-215
HOPX Rabbit IgG Proteintech - #11419-1-AP
HOPX Mouse IgG1 Santa Cruz - #sc-398703
Iba1 Rabbit IgG Wako - 019-19741
Ki67 Rabbit IgG Abcam - #Ab15580
Nestin Rabbit IgG Abcam - #Ab27952
NeuN Mouse IgG1 Merck - #MAB377
Olig-2 Rabbit IgG Merck - AB9610
PTPRZ1 Rabbit IgG Sigma - #HPA015103
Reelin Mouse IgG1 Merck - #MAB5366
S100β Mouse IgG1 Merck - S2532
SATB2 Rabbit IgG Abcam - #ab34735
Sox2 Rat IgG Invitrogen - #14-9811-82
Sox2 Mouse IgG1 Abcam #Ab79351
MAP2 Chicken IgY Abcam - #ab5392
CD31 Rat IgG2a, κ BioLegend - #102513
Anti-Heparan Sulfate Proteoglycan (Perlecan) Antibody, clone A7L6 Rat IgG2ak Sigma-Aldrich - #MAB1948P
BCAN Rabbit IgG Proteintech - #19017-1-AP
Anti-Chondroitin Sulfate antibody [CS-56] Mouse IgM Abcam - #ab11570
COL3A Rabbit IgG Proteintech - 22734-1-AP
COLIVa1 Rabbit IgG NOVUS - NB120-6586
PDGFRB Rabbit IgG Invitrogen MA5-15143
IL17RA Rabbit IgG Invitrogen PA5-34571
IL17RC Rabbit IgG Invitrogen PA5-54108
ACT1 Rat IgG1 Invitrogen 14-4040-82
phospho-P65 Rabbit IgG Abcam ab28856
TRAF6 Rabbit IgG Invitrogen 702286
Alexa Fluor 555 Donkey ms-IgG (H+L) anti mouse Invitrogen - A-31570
Alexa Fluor 488 Goat rat-IgG (H+L) anti Rat Invitrogen - A-11006
Alexa Fluor 594 Goat rat-IgG (H+L) anti Rat Invitrogen - A-11007
Alexa Fluor 647 Goat rat-IgG (H+L) anti Rat Invitrogen - A-21247
Alexa Fluor 488 Donkey sh-IgG (H+L) anti sheep Invitrogen - A-11015
Alexa Fluor 488 Goat mouse-IgG (H+L) anti mouse Invitrogen - 10727464
Alexa Fluor 647 Goat mouse-IgG (H+L) anti Rabbit Invitrogen - 15627898
Alexa Fluor 488 Goat rabbit-IgG (H+L) anti Rabbit Invitrogen - 10236882
Alexa Fluor 594 Goat rabbit-IgG (H+L) anti Rabbit Invitrogen - 10266352
Alexa Fluor 647 Goat rabbit-IgG (H+L) anti Rabbit Invitrogen - 15615485
Alexa Fluor 594 Goat mouse-IgG (H+L) anti mouse Invitrogen - 10524773

Alexa Fluor 488 Goat chicken-IgY (H+L) anti Chicken Invitrogen - 10286672

Validation

Validations were conducted based on the manufacturer's websites or previously published work. Primary antibodies used in this study were selected based on established specificity and prior use in the literature. The number of independent publications reporting each antibody is provided:
BM28/MCM2 (BD Biosciences #610701) — 39 publications
Cleaved Caspase-3 (CST #9661) — 10,478 publications
CTIP2 (Abcam #ab18465) — 841 publications
EOMES/TBR2 (R&D Systems #AF6166) — 25 publications
GFAP (Millipore #MAB360) — 65 publications
GFAP (Merck #AB5541) — 299 publications
GFP (Rockland #600-101-215) — 419 publications
HOPX (Proteintech #11419-1-AP) — 36 publications
HOPX (Santa Cruz #sc-398703) — 81 publications
Iba1 (Wako #019-19741) — 4,528 publications
Ki67 (Abcam #ab15580) — 4,218 publications
Nestin (Abcam #ab27952) — 20 publications
NeuN (Merck #MAB377) — 6,946 publications
Olig2 (Merck #AB9610) — 1,170 publications
PTPRZ1 (Sigma #HPA015103) — 6 publications
Reelin (Merck #MAB5366) — 68 publications
S100β (Merck #S2532) — 386 publications
SATB2 (Abcam #ab34735) — 90 publications
SOX2 (Invitrogen #14-9811-82) — 67 publications
SOX2 (Abcam #ab79351) — 101 publications
MAP2 (Abcam #ab5392) — 1,135 publications
CD31 (BioLegend #102513) — 26 publications
Perlecan (Sigma #MAB1948P) — 46 publications
BCAN (Proteintech #19017-1-AP) — 2 publications
Chondroitin sulfate (Abcam #ab11570) — 72 publications
COL3A (Proteintech #22734-1-AP) — 381 publications
COLIVα1 (Novus #NB120-6586) — 39 publications
PDGFRβ (Invitrogen #MA5-15143) — 11 publications
IL17RA (Invitrogen #PA5-34571) — 3 publications
ACT1 (Invitrogen #14-4040-82) — 5 publications
phospho�p65 (Abcam #ab28856) — 77 publications
TRAF6 (Invitrogen #702286) — 1 publication

Eukaryotic cell lines

Policy information about cell lines and Sex and Gender in Research

Cell line source(s)

KOLF2.1J iPSC - The Jackson Laboratory, JIPSC001000
Human iPSC line SCTi003-A—derived neural progenitor cells (NPCs) (Stemcell Technologies, 200-0620)

Authentication

Authentication was performed by the companies.

Mycoplasma contamination

According to the company's report, all cells tested negative for mycoplasma contamination. Since we performed the experiments immediately after obtaining the cell lines from the company, we did not conduct a mycoplasma contamination test in the lab.

Commonly misidentified lines
(See ICLAC register)

No commonly misidentified lines were used.

Plants

Seed stocks

n/a

Novel plant genotypes

n/a

Authentication

n/a
